# Darolutamide antagonizes androgen signaling by blocking enhancer and super‐enhancer activation

**DOI:** 10.1002/1878-0261.12693

**Published:** 2020-06-05

**Authors:** Simon J. Baumgart, Ekaterina Nevedomskaya, Ralf Lesche, Richard Newman, Dominik Mumberg, Bernard Haendler

**Affiliations:** ^1^ Research and Development, Pharmaceuticals Bayer AG Berlin Germany

**Keywords:** androgen receptor, cistrome, FOXA1, histone acetylation, prostate cancer, super‐enhancer

## Abstract

Prostate cancer (PCa) is one of the most frequent tumor types in the male Western population. Early‐stage PCa and late‐stage PCa are dependent on androgen signaling, and inhibitors of the androgen receptor (AR) axis represent the standard therapy. Here, we studied in detail the global impact of darolutamide, a newly approved AR antagonist, on the transcriptome and AR‐bound cistrome in two PCa cell models. Darolutamide strongly depleted the AR from gene regulatory regions and abolished AR‐driven transcriptional signaling. Enhancer activation was blocked at the chromatin level as evaluated by H3K27 acetylation (H3K27ac), H3K4 monomethylation (H3K4me1), and FOXA1, MED1, and BRD4 binding. We identified genomic regions with high affinities for the AR in androgen‐stimulated, but also in androgen‐depleted conditions. A similar AR affinity pattern was observed in healthy and PCa tissue samples. High FOXA1, BRD4, H3K27ac, and H3K4me1 levels were found to mark regions showing AR binding in the hormone‐depleted setting. Conversely, low FOXA1, BRD4, and H3K27ac levels were observed at regulatory sites that responded strongly to androgen stimulation, and AR interactions at these sites were blocked by darolutamide. Beside marked loss of AR occupancy, FOXA1 recruitment to chromatin was also clearly reduced after darolutamide treatment. We furthermore identified numerous androgen‐regulated super‐enhancers (SEs) that were associated with hallmark androgen and cell proliferation‐associated gene sets. Importantly, these SEs are also active in PCa tissues and sensitive to darolutamide treatment in our models. Our findings demonstrate that darolutamide is a potent AR antagonist blocking genome‐wide AR enhancer and SE activation, and downstream transcription. We also show the existence of a dynamic AR cistrome that depends on the androgen levels and on high AR affinity regions present in PCa cell lines and also in tissue samples.

## Graphical abstract

Schematic model showing how darolutamide blocks androgen (R1881)‐mediated AR signaling by inhibiting the function of normal enhancers and super‐enhancers, and impairing downstream gene transcription.

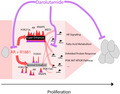

**Abbreviations**
AR: androgen receptor
FDR: false discovery rate
GSEA: gene set enrichment analysis
NE: normal enhancer
PCa: prostate cancer
ROSE: ranking of super‐enhancers
SE: super‐enhancer

## 1 Introduction

The androgen receptor (AR) is a clinically validated target for the treatment of early‐ and late‐stage prostate cancer (PCa). Androgen deprivation therapy (ADT) represents the preferred initial pharmaceutical treatment for PCa and is used for nearly half the men with this disease (Gilbert *et al*., 2011). Castration resistance often follows and AR inhibitors that lower androgen levels or directly block the AR (e.g., AR antagonists) have proven highly beneficial (Miura and Horie, 2019; Nevedomskaya *et al*., 2018; Pagliuca *et al*., 2019). Improved outcomes for the combination of ADT with AR inhibitors have recently been shown in pivotal clinical studies both for nonmetastatic and metastatic PCa (Cattrini *et al*., 2019; Hess‐Busch *et al*., 2019; Sathianathen *et al*., 2019). Therapy resistance usually takes place after some time, but multiple studies show that the AR remains the main driver also in these late‐stage patients. Indeed, a majority of metastatic PCa lesions have amplification of the AR gene and/or of an upstream regulatory enhancer element (Quigley *et al*., 2018; Robinson *et al*., 2011; Takeda *et al*., 2018; Viswanathan *et al*., 2018). Also, the levels of ligands are often increased, so that tumor cells remain exposed to residual androgen action (Scher and Sawyers, 2005). This underscores the unique importance of the AR and its downstream transcriptome in this disease so that understanding in detail the molecular mode of action of antagonist treatment will help toward future treatments. Little is known about how AR antagonists act on the genome‐wide AR binding (AR cistrome), the local chromatin environment, or cofactor occupancy.

Androgen‐stimulated AR binds as a homodimer to cognate response elements found in the genome (Claessens *et al*., 2017) and interacts with a number of cofactors (Liu *et al*., 2017; Shiota *et al*., 2011). This allows the recruitment of transcription factors to promoter and enhancer regions, and the downstream regulation of gene expression (Baumgart *et al*., 2019; Toropainen *et al*., 2016; Wilson *et al*., 2016). AR signaling has an essential role in the normal male physiology but also fuels growth, proliferation, and metastasis of PCa (Wang *et al*., 2007; Wang *et al*., 2009; Xu *et al*., 2006). This malignant role is due to an aberrant AR cistrome found in tumor cells which activates downstream cancerous pathways (Armenia *et al*., 2018; Copeland *et al*., 2019; Pomerantz *et al*., 2015; Wang and Koul, 2017; Wang *et al*., 2009). AR‐controlled enhancers are marked by histone acetylation, H3K4 monomethylation, and binding sites for AR itself as well as dedicated coregulators such as FOXA1 (Taslim *et al*., 2012). Recently, a novel class of enhancers called super‐enhancers (SEs) has been defined (Loven *et al*., 2013; Vaharautio and Taipale, 2014; Whyte *et al*., 2013). SEs strongly regulate oncogenes and cell identity genes, and are activated in a tumor‐ and cell‐type‐specific manner. They are highly occupied by lineage‐specific transcription factors, by the mediator complex protein MED1, and by the bromodomain protein BRD4 and are also characterized by much elevated histone acetylation. A role for aberrant SE activity in different tumor types has been reported in recent studies (Bao *et al*., 2019; Chapuy *et al*., 2013; van Groningen *et al*., 2017; Loven *et al*., 2013; Natsume *et al*., 2019; Zanconato *et al*., 2018). Targeting SE‐associated factors has a strong impact on the transcriptional output of the associated cancer genes and subsequently on cell proliferation, resistance mechanisms, and cell identity, implying that compounds that interfere with SE function may have unique anticancer properties.

Darolutamide is a novel AR antagonist recently approved for nonmetastatic castration‐resistant PCa patients by the American Food and Drug Administration (Fizazi *et al*., 2019). Darolutamide binds with high affinity to the ligand‐binding pocket of the AR and efficiently blocks homodimerization of the AR (Moilanen *et al*., 2015; Sugawara *et al*., 2019). It inhibits AR translocation into the nucleus, thus leading to reduction of androgen target gene expression and ultimately decreasing proliferation of PCa cells (Moilanen *et al*., 2015). This translates into high antitumor efficacy *in vivo* for different cell line‐ and patient‐derived PCa models (Borgmann *et al*., 2018; Moilanen *et al*., 2015; Sugawara *et al*., 2019). Genome‐wide studies on the effects of darolutamide on AR signaling have not been performed yet and would give novel insights into the regulatory network affected by this novel compound.

In this work, we studied the effects of darolutamide on the AR cistrome, on selected AR transcriptional coregulators and enhancer‐associated histone modifications. We found that darolutamide strongly antagonized genome‐wide AR binding and inhibited androgen‐dependent gene regulation. We identified different AR‐interacting genomic regions with varying AR recruitment patterns after androgen induction and with different sensitivities to darolutamide. Interestingly, darolutamide decreased binding of the essential pioneer factor FOXA1 at AR‐binding sites. SEs were also identified in cellular PCa models and matched to PCa tissues. These SEs were strongly affected by darolutamide treatment and associated with hallmark AR signaling as well as cell proliferation gene signatures. Overall, this study describes in detail the genome‐wide antagonistic mechanism of action of darolutamide on AR and its downstream effects on the enhancer landscape and on regions with different AR affinities.

## 2 Material and methods

### 2.1 Cell culture and reagents

VCaP and LAPC4 cells were purchased from the American Type Culture Collection (ATCC, Manassas, VA, USA) and the Deutsche Sammlung von Mikroorganismen und Zellkulturen (DSMZ, Braunschweig, Germany). Cells were routinely cultured as described previously (Sugawara *et al*., 2019; Sugawara *et al*., 2016). They were stimulated with the synthetic androgen R1881 at a concentration of 1 nm after 2 days of starvation in medium supplemented with 10% charcoal‐stripped FBS. Darolutamide was added at a final concentration of 500 nm (low) or 2 µm (high), and the cells were harvested 8 or 22 h post‐treatment.

### 2.2 RNA isolation and sequencing

Cells were lyzed and RNA was isolated using RNeasy columns with on‐column DNA digestion, as described by the manufacturer (Qiagen, Hilden, Germany). RNA integrity was measured, and samples with values above eight were further processed. RNA library preparation was performed after mRNA purification using poly‐T beads, as described by the manufacturer (TruSeq Stranded mRNA Kit; Illumina, San Diego, CA, USA). Five biological replicates per condition were sequenced on a hiSeq2500 device via single‐end, 50 base‐pair reads with an average depth of 21 million reads per sample (Illumina, HiSeq2500 HTv4, SR, dual‐indexing, 50 cycles).

### 2.3 RNA‐seq bioinformatics analysis

FASTQ reads were mapped via STAR aligner to the human genome GRCh38 and quantified with featureCounts from the Subread package (Liao *et al*., 2014). Genes with at least 10 reads in four samples or more were used for further analysis (*N* = 19 276 in VCaP and *N* = 18 310 in LAPC4). Differentially expressed genes were identified with DESeq2 (Love *et al*., 2014). Gene set enrichment analysis (GSEA) (Subramanian *et al*., 2005) was performed with default parameters on preranked datasets sorted by log_2_FC. Differentially regulated genes were defined as having adjusted *P*‐values lower than 0.05 and absolute log_2_‐fold values higher than one, if not stated otherwise. Raw and processed data are available at NCBI GEO (https://www.ncbi.nlm.nih.gov/geo/) under GSE148397. Gene expression differences of SE and normal enhancer (NE) gene groups were compared by a two‐sample *t*‐test.

### 2.4 ChIP‐sequencing experiments

For ChIP experiments with subsequent sequencing (ChIP‐seq), three replicates of eight million cells were seeded in 15‐cm plates and treated with 2 µm darolutamide plus 1 nm R1881 for 22 h, subsequently fixed for 10 min with 1% formaldehyde, and processed as described previously (Baumgart *et al*., 2017). For each ChIP reaction, three million cells were used and probed with the mentioned antibodies (Table S1). Efficiency and specificity of ChIPs were evaluated by ChIP‐qPCR. ChIP experiments were performed in biological triplicates, and library preparation was done as described by the manufacturer (MicroPlex Library Preparation Kit v2; Diagenode SA, Seraing, Belgium). The libraries were sequenced on a HiSeq2500 Illumina machine with 50 base‐pair, single‐end reads to an average depth of 25–30 million reads per sample. For ChIP‐seq experiments performed in collaboration with Diagenode, cells were fixed for 10 min with 1% formaldehyde before shipment. ChIP was performed according to the iDeal ChIP‐seq kit for histones or transcription factors (Diagenode) with subsequent ChIP‐qPCR testing and library preparation as described above.

### 2.5 ChIP‐seq bioinformatics analysis

Raw and processed data are available at NCBI GEO (https://www.ncbi.nlm.nih.gov/geo/) under GSE148358. Sequencing reads were mapped to human genome hg19 using the Burrows–Wheeler alignment tool with default settings (Li and Durbin, 2009). Duplicate reads were marked with Picard tools and filtered out together with multimapping reads (MAPQ score below 30). Narrow peaks were called by MACS2 (Zhang *et al*., 2008) with default parameters and a *q*‐value cutoff of 0.05. Human genome blacklisted regions (ENCODE consortium) were excluded from further analysis. Peaks present in at least two replicates were used for further analysis. BAM files were used individually or merged and converted to bigwig format via Deeptools2 bamCoverage with default parameters and reads per kilobase of transcript per million mapped reads normalization (Ramirez *et al*., 2016). For visualization, the plotProfile, plotHeatmap, and plotCorrelation (Pearson) programs were used with computeMatrix or multiBigwigSummary outputs from bigwig files. SEs were identified using the ranking of super‐enhancers (ROSE) algorithm (Hamdan and Johnsen, 2018; Loven *et al*., 2013; Whyte *et al*., 2013) with default parameters and exclusion of transcription start site (TSS) regions (+/− 2.5 kb) using MED1 or H3K27ac signals as quantitative measures in VCaP and LAPC4 cells, respectively. AR peak regions called in R1881‐treated conditions were used as identifiers for SE regions. These SE regions were scaled to an average size of 12.3 kbp via computeMatrix scale‐regions for VCaP cells, and average signals were computed and plotted. Single genomic regions were visualized with Integrated Genome Viewer (Robinson *et al*., 2011; Thorvaldsdottir *et al*., 2013). The bigwig ChIP‐seq data files of H3K27ac (GSE56288) (Pomerantz *et al*., 2015) and AR (GSE96652) (Kron *et al*., 2017) originating from patients were processed as outlined above. The FOXA1 and BRD4 ChIP‐seq files were from GSE123625 (SRR8311364) (Parolia *et al*., 2019) and GSE55062 (Asangani *et al*., 2014), respectively. Genes associated with SEs or NEs were identified by overlapping gene bodies plus a region 50 kbp upstream of the TSS. GREAT analysis (version 3.0.0) was performed for identification of gene sets close to the indicated bed regions used as input with the default parameters (McLean *et al*., 2010). Top five enriched terms are shown. Motif analysis was performed using the SeqPos motif tool with default settings (Liu *et al*., 2011).

## 3 Results

### 3.1 Darolutamide blocks transcriptional response to androgen signaling

**Fig. 1:**
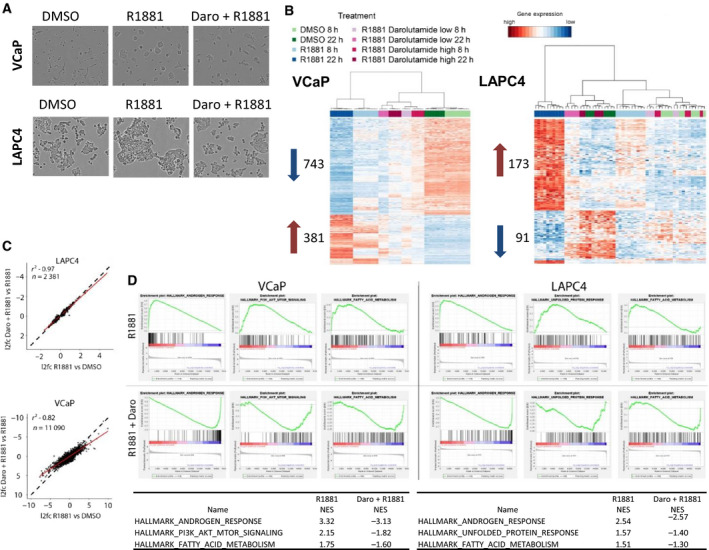
Darolutamide blocks the AR‐mediated transcriptional response to R1881. (A) Microscopic analysis of VCaP and LAPC4 cell morphology after 72 h treatment with DMSO, 1 nm R1881, or 1 nm R1881 plus 2 µm darolutamide under 10x magnification. (B) Heatmap of R1881‐regulated genes in VCaP (left) and LAPC4 (right) cells. Genes (rows) and treatments (columns) are ordered by unsupervised hierarchical clustering. Genes were selected by abs(log_2_FC)>2 and adjusted *P* < 0.01, and abs(log_2_FC)>0.5 and adjusted *P* < 0.25 in VCaP and LACP4 cells, respectively. Cells were treated for 8 or 22 h with DMSO, 1 nm R1881, or 1 nm R1881 plus 500 nm (low) or 2 µm (high) darolutamide. The number of up‐ and down‐regulated genes is indicated by red and blue arrows, respectively. (C) Scatter plot of log_2_fold changes (l2fc) in gene expression comparing genes regulated by R1881 (*x*‐axis) and by R1881 plus 2 µm darolutamide (*y*‐axis). Linear regression fit (red line) with the respective residual^2^ (*R* ^2^) value determined for LAPC4 and VCaP cells for R1881 plus 2 µm darolutamide‐regulated genes vs R1881 at the 22 h time point. The dotted lines show a perfect fit with *x* = *y*. (D) Gene set enrichment analysis: enrichment of hallmark gene sets among the genes regulated by treatment with R1881 vs R1881 plus 2 µm darolutamide in VCaP and LAPC4 cells at the 22 h time point. Normalized enrichment scores are shown.

Following treatment of R1881‐stimulated VCaP or LAPC4 cells with the AR antagonist darolutamide, we observed decreased proliferation and changed morphology compared to R1881 treatment alone, as expected (Sugawara *et al*., 2019) (Fig. 1A). For a detailed characterization of the underlying effects of darolutamide on the PCa transcriptome, we analyzed RNA samples from both cell lines following stimulation with 1 nm R1881 and treatment with two darolutamide concentrations, 500 nm (low) and 2 µm (high), and for two timespans (8 and 22 h) in each case. The concentrations selected corresponded to the IC50 and IC90 values previously determined in cell proliferation assays (Sugawara *et al*., 2019). First, we analyzed the transcriptional changes induced by the compounds using unsupervised, hierarchical clustering. The samples clustered accordingly to treatments and time points (Fig. 1B). Notably, the R1881‐ plus darolutamide‐treated condition clustered together with the DMSO condition, implying that antagonist treatment comprehensively reverted the effects elicited by androgen. The antagonistic effects of darolutamide were dose‐dependent and visible at the early and late time points, demonstrating a rapid and durable block of AR signaling. Next, we directly compared the expression of the significantly R1881‐regulated genes (*P*‐adj < 0.05) with that of the darolutamide‐ plus R1881‐regulated genes. Altogether there were less genes up‐ or down‐regulated by R1881 in LAPC4 cells than in VCaP cells (Fig. 1B). In LAPC4 cells, there was an excellent linear negative correlation between almost all R1881‐regulated genes and those blocked by darolutamide, as indicated by the linear model fit (red line) (Fig. 1C). In VCaP cells, there were almost five times more R1881‐regulated genes, and here also, a strong block by darolutamide of the R1881‐mediated gene expression was observed. The negative correlation showed that most R1881‐regulated genes were blocked by darolutamide. The correlation was not as strong as in LAPC4 cells, and there were examples of genes with different sensitivities to antagonist treatment (Fig. 1C). GSEA revealed that in both cell lines the ‘hallmark androgen response’ gene set was the most enriched one when comparing R1881 alone to R1881 plus darolutamide treatment (Fig. 1D). In addition, genes from the fatty acid metabolism pathway, which plays a unique role in PCa (Dang *et al*., 2019), were also regulated in both cell lines. Genes from the unfolded protein response were negatively affected by darolutamide in LAPC4 cells (Sheng *et al*., 2015) (Fig. 1D). PI3K‐AKT‐mTOR signaling, which is critical for PCa cell proliferation (Jamaspishvili *et al*., 2018), was negatively affected by darolutamide in VCaP cells (Fig. 1D). When considering all time points, concentrations, and cell lines tested, the hallmark androgen response gene set was the most consistently and strongly enriched one after darolutamide treatment (Fig. S1, Tables S2‐S9). Altogether these data show that darolutamide efficiently blocked the transcriptional effects elicited by R1881 treatment, in particular those linked to the androgen response pathway.

### 3.2 Darolutamide inhibits androgen‐induced changes at the AR cistrome

**Fig. 2:**
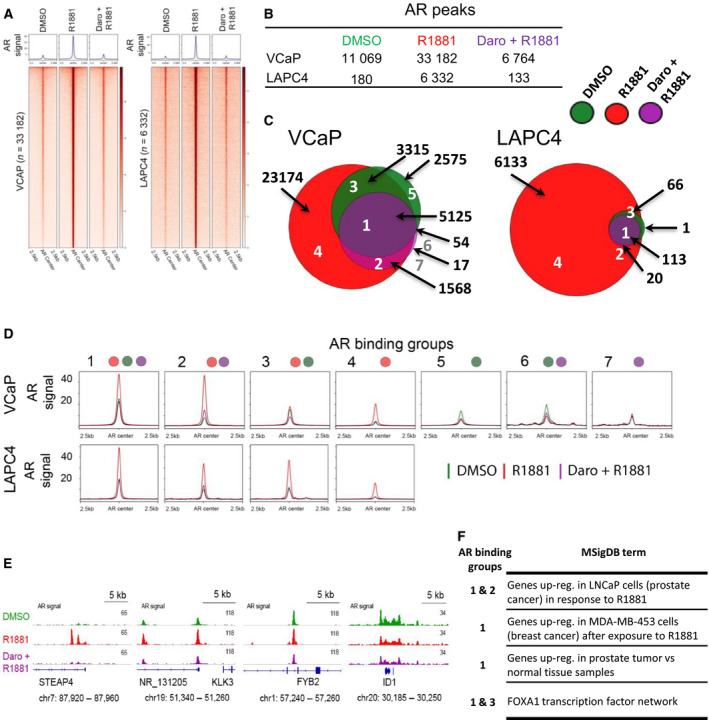
Genome‐wide AR occupancy is reduced by darolutamide treatment. (A) AR ChIP‐seq signals are shown at R1881‐induced AR‐binding sites for cells treated with DMSO, R1881, or R1881 plus 2 µm darolutamide. Regions were ordered by descending signal intensity of R1881 samples for each cell line. Number of binding sites and averaged signal at centered AR regions plus/minus 2.5 kbp are shown. (B) Table with number of AR peaks and (C) Euler diagram with overlapping AR peaks for the indicated conditions in VCaP (left) and LAPC4 cells (right). (D) Mean AR signals of DMSO (green), R1881 (red), and R1881 plus 2 µm darolutamide (purple) conditions at the identified AR‐binding clusters. (E) ChIP‐seq signals at the indicated gene regions for the different treatment conditions. (F) Selection of top enriched gene sets from Molecular Signaling Database associated with AR clusters defined in VCaP cells and analyzed by GREAT. All sets identified have a false discovery rate (FDR) below 0.05. Full tables are shown in Table S11.

For a better understanding of the regulatory mechanisms underlying the transcriptional blockade of androgen action by darolutamide, we analyzed the AR cistrome in VCaP and LAPC4 cells treated for 22 h with the 2 µm darolutamide concentration by performing ChIP‐seq with an AR‐specific antibody. A strong, genome‐wide gain of AR binding was observed for the R1881 condition, and this was entirely reversed to the DMSO control level when combining with darolutamide (Figs 2A and Fig. S2A). In total, we identified 33 182 AR peaks in VCaP cells and 6332 AR peaks in LAPC4 cells after R1881 stimulation (Fig. 2B). This corresponded well with the stronger response observed at the transcriptional level upon R1881 treatment (Fig. 1B) and is possibly linked to the AR gene amplification and consequently the higher AR protein levels present in VCaP compared to LAPC4 cells (Sugawara *et al*., 2016). We then looked at AR binding in hormone‐depleted and darolutamide‐treated conditions. The number of AR peaks in the R1881 plus darolutamide group was lower than in the DMSO control condition in both cell lines, showing that the compound was able to diminish residual AR binding present in hormone‐depleted medium (Fig. 2C). A few overlapping AR peaks were observed in VCaP cells, but not in LAPC4 cells, in the DMSO and R1881 plus darolutamide conditions. Concerning the peaks identified in the R1881 plus darolutamide condition, there was an overlap with the peaks found in the DMSO and in the R1881 conditions, implying that a subset of AR‐bound regions was occupied constitutively without androgen treatment and therefore not affected by AR antagonist treatment. Most of these constitutive peaks were found in VCaP cells, and we assigned them to 2455 genes based on location 20 kbp upstream of the TSS and in gene‐coding regions. In order to find out whether longer treatment times than those we used affected this pattern, we looked at publicly available datasets. We found no adequate comparator for the AR cistrome; however, the transcriptional changes in LNCaP cells during 12 months of androgen deprivation have been reported in GSE8702 (D'Antonio *et al*., 2008). When looking at the 2455 genes, we found that only 12% of them displayed a significant difference in expression following long‐term androgen deprivation (ANOVA *P*‐value adjusted for multiple testing < 0.05; data not shown). Interestingly, in VCaP cells, there were 2575 AR peaks present in the DMSO‐only condition that were not seen in the R1881 condition, suggesting that androgen treatment can redistribute part of the AR cistrome (Fig. 2C). When we compared the AR cistromes between both cell lines, we found that the highest overlap was in regions with AR binding in all conditions (Fig. S2B). For the 86 common peaks, we identified 50 corresponding genes, based on the location of these peaks either within 20 kbp upstream of the TSS or in the gene body (Table S10). The expression of these genes was not strongly modulated in VCaP and LAPC4 cells upon treatment with androgen and darolutamide (not shown). Gene knock‐out data are available for 47 of these genes in the public Dependency Map portal (Tsherniak *et al*., 2017), and very few had an impact on cell viability (not shown). The second most abundant overlap was in the R1881‐only treated group (Fig. S2B). The other groups did not show an overlap, indicating that the AR cistromes differed to some extent between the VCaP and LAPC4 cells.

In order to further characterize the AR‐bound regions in relationship to the different conditions, we defined groups based on individual treatments and their peak overlaps and depicted the respective averaged AR‐binding intensities for each of them (Fig. 2D). The highest AR binding upon R1881 induction was observed in groups 1 (peak overlap between all three conditions) and 2 (peaks in R1881‐treated and R1881‐ plus darolutamide‐treated conditions), followed by groups 3 (DMSO‐ and R1881‐treated overlapping peaks) and 4 (peaks unique to R1881‐treated samples) in both VCaP and LAPC4 cells. Groups 1–4 showed the strongest decrease in AR binding when comparing the R1881 to the R1881 plus darolutamide condition. Groups 5 (peaks unique to DMSO‐treated samples) and 6 (peak overlap between DMSO‐treated and R1881‐ plus darolutamide‐treated samples) had on average lower AR signal levels in the R1881 condition and were only identified in VCaP cells. In both these groups, a higher averaged AR binding was measured in the DMSO condition in comparison with the R1881‐ and darolutamide‐treated conditions. Group 7 (peaks in R1881‐ plus darolutamide‐treated samples) was also only identified in VCaP cells and comprised just 17 AR‐bound regions with low, comparable AR‐binding levels for all treatments (Fig. 2C). Overall, we found that AR‐interacting regions varied in number, location, and binding intensity which suggests a very dynamic AR cistrome among all conditions. Redistribution of AR binding was also observed at the individual gene level (Fig. 2E). Interestingly, some regions had a higher AR retention in hormone‐depleted and darolutamide plus R1881 condition than in the R1881 condition, implying variance in sensitivity of AR depletion from the chromatin depending on the region. Altogether, this further underlines that darolutamide efficiently blocked AR binding and did not lead to novel AR peaks on the genome. We additionally compared the identified AR‐binding groups in VCaP cells to AR occupancy in healthy and malignant prostate samples to determine whether a similar pattern also existed in patients. Relative AR‐binding intensities in healthy and cancerous prostate tissues were very similar to the AR binding observed in VCaP cells, with groups 1 and 2 showing the highest levels. This suggests that overarching high AR affinity regions which maintain AR binding throughout benign and malignant tissues, probably also after treatment regimens, exist in patients (Fig. S3).

Next, we used GREAT analysis to identify genes associated with the AR‐binding groups and determine their potential role in biological pathways. Focusing on VCaP cells, we found that regions from groups 1 and 2 which had the highest AR signals in R1881‐treated conditions were proximal to genes responsive to R1881 (Fig. 2F, Table S11). Groups 1 and 3 were associated with the FOXA1 transcription factor network suggesting that this pioneer factor maintained AR accessibility at these regions, which might be needed to sustain AR occupancy under low‐androgen condition (Fig. 2F, Table S11). Interestingly, group 2 which also gained massive AR binding following R1881 treatment was not enriched for FOXA1 network genes, implying a distinct regulatory mechanism. Group 5 had higher AR binding in the DMSO than in the R1881 condition and was significantly enriched in gene sets involved in signal transduction, in ERK activation, and in PI3K/AKT/mTOR signaling events (Table S11). This might represent an alternative growth mechanism for cells exposed to low‐androgen concentrations. The other groups had too few AR‐bound regions to allow the identification of significantly enriched pathways.

To further elaborate on the binding and affinity mechanisms of the different AR sites, we performed a detailed motif analysis. Overall, we detected a high over‐representation of binding motifs for forkhead domain family, homeodomain family, and hormone‐nuclear receptor family proteins, including motifs for oncogenic factors known to play a role in PCa such as FOXA1, Nkx3‐1, and GATA2 (Fig. S2C). In VCaP cells, differences in motif enrichment were observed between groups 1, 2, and 4 which had higher enrichment of androgen response elements compared to groups 3 and 5, and this fitted with the increase in AR interaction observed upon androgen treatment. In group 5 with constitutive AR binding, an enrichment of the GATA domain family motif was observed. Group 3 did not show a striking enrichment apart from forkhead box protein motifs. Concerning LAPC4 cells, only group 4 was large enough for robust analysis and the pattern looked similar to that of group 4 from VCaP cells with highest enrichments of nuclear hormone receptor and forkhead domain family motifs (Fig. S2C).

In summary, darolutamide treatment reduced AR binding globally and no novel AR‐binding sites were identified. Regions displaying AR occupancy under hormone‐depleted condition or after antagonist treatment were found, and this constitutive interaction might rely to a strong extent on cobinding DNA‐interacting proteins that compensate for the comparatively weak AR binding.

### 3.3 Darolutamide blocks enhancer activation

**Fig. 3:**
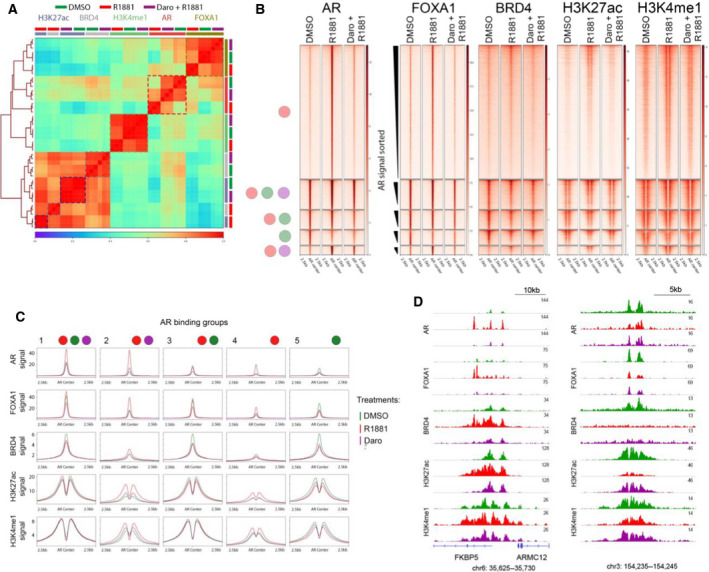
AR enhancer activation is reverted by darolutamide. (A) Heatmap of Pearson’s correlation sorted by unsupervised, hierarchical clustering of listed ChIP‐seq data from cells treated with DMSO, R1881, or R1881 plus 2 µm darolutamide. (B) Heatmaps of averaged ChIP‐seq signals for FOXA1, BRD4, H3K27ac, and H3K4me1 at the AR‐binding clusters described in Fig. 2B for DMSO, R1881, or R1881 plus 2 µm darolutamide samples. Regions are sorted from high to low AR signals in the R1881 condition. (C) Averaged signals of ChIP‐seq samples from different treatment conditions are shown at AR‐binding sites for different groups. (D) ChIP‐seq signals of different binding proteins and histone modifications at single genomic loci after the mentioned treatments. Scales are shown at the top right of each panel.

DNA accessibility and the chromatin environment governed by local histone modifications play a major role in gene transcription control of AR signaling. To better understand why certain genome regions have higher AR affinity than others and in which chromatin context AR binding is most strongly affected by darolutamide, we performed additional ChIP‐seq experiments in VCaP cells. The levels of the histone modifications H3K27ac and H3K4me1 which are markers of active enhancers (Catarino and Stark, 2018; Creyghton *et al*., 2010), and binding of the enhancer‐associated factors FOXA1 and BRD4 were analyzed after DMSO, R1881, or R1881 plus darolutamide treatment. We performed correlation analysis of the signal of different marks 1 kb around all AR‐binding sites. The replicates for the different conditions clustered together and showed the highest correlations to each other, indicating high reproducibility (Fig. 3A). Generally, a stronger correlation was observed between the DMSO and R1881 plus darolutamide groups, compared to the R1881 group. Marked differences were observed for AR, FOXA1, BRD4, and H3K27ac between the R1881‐treated groups and the other treatments. Conversely, H3K4me1 levels were not very different between the treatment groups. We next focused on the AR‐binding groups with over 60 regions and therefore excluded groups 6 and 7 which comprised 17 and 54 regions, respectively. We plotted the signal of each factor or histone modification sorted by the descending AR occupancy previously determined in the R1881 treatment group (Figs 3B and Fig. S4A). Generally, the signal intensities of the marks increased with AR occupancy, but to different extents. The overall pattern was similar between the treatment groups, indicating there were no massive rearrangements in the binding intensities between treatments. The top enriched regions remained similar between the treatments, but the signal intensities varied. Similar findings were made in LAPC4 cells (Fig. S4B). Only minor differences were observed for H3K4me1 in the defined AR‐binding groups. We furthermore looked at H3K18ac and H2BK15ac, two marks preferentially acetylated by p300/CBP, a histone acetyltransferase implicated in maintenance of PCa proliferation (Damodaran *et al*., 2017; Ianculescu *et al*., 2012; Jin *et al*., 2017; Weinert *et al*., 2018) and observed patterns similar to that of H3K27ac at AR‐bound regions (Fig. S4C).

### 3.4 Darolutamide depletes AR and FOXA1 from enhancer

Next, we evaluated the averaged signals obtained for the different marks in each of the defined groups (Fig. 3C). Strong FOXA1 signals corresponded to high AR signals in each respective treatment condition. This fitted well with the high correlation observed before in unsupervised analysis (Fig. 3A). On average, the strongest gains of FOXA1 after R1881 treatment compared to DMSO were observed in groups 2 and 4. As both the FOXA1 and AR gains were blocked with darolutamide, it is likely that FOXA1 binding was enabled by AR binding (Zhao *et al*., 2016). This was also observed at the single gene level (Fig. 3D). The groups 1, 3, and 5 which showed AR binding already in the DMSO condition also had very high FOXA1 signals (Fig. 3C), implying that high occupancy of FOXA1 also contributed to high AR occupancy, which is in line with a previously described role of FOXA1 in recruiting AR (Zhao *et al*., 2014). This was also observed when comparing the single replicates over all regions in the respective groups (Fig. S4A) and at the single gene level (Fig. 3D). To further test whether regions with AR occupancy already in the DMSO condition also showed higher FOXA1 occupancy in another cell line, we used publicly available FOXA1 ChIP‐seq data from LAPC4 cells not treated with androgen and plotted the signals against the identified AR groups. Indeed, we observed high FOXA1 levels in the groups with AR occupancy in the DMSO condition, which supports the idea that residual AR binding under low‐androgen condition was associated with high levels of FOXA1 (Fig. S5A). Beside FOXA1 binding, we also observed high BRD4 binding in groups 1, 3, and 5, and this was further confirmed by examining BRD4 ChIP‐seq data from VCaP cells originating from another study (Fig. S5B). The highest average difference between the R1881 and the R1881 plus darolutamide conditions with regard to BRD4 binding was observed in groups 2 and 4. In both cases, darolutamide lowered BRD4 occupancy and this was also observed at the single gene level (Fig. 3D). The strongest changes in the histone modifications H3K27ac and H3K4me1 were again observed in the groups 2 and 4, and this was also blocked by darolutamide. It is noteworthy that groups 1, 3, and 5 were highly primed by H3K4me1, H3K27ac, and BRD4 before R1881 induction, and consequently, these patterns were not strongly altered by darolutamide treatment. The situation was similar for H2BK15 and H3K18 acetylation in these groups (Fig. S4C). This suggests that regions with high acetylation and high binding by activating factors were on average less affected at the chromatin level by low‐androgen levels or by AR antagonist treatment.

Overall, these results indicate that darolutamide remodeled the AR and FOXA1 cistromes with a stronger impact on sites being activated by androgen than at sites that were already primed.

### 3.5 Darolutamide decreases super‐enhancer activity

**Fig. 4:**
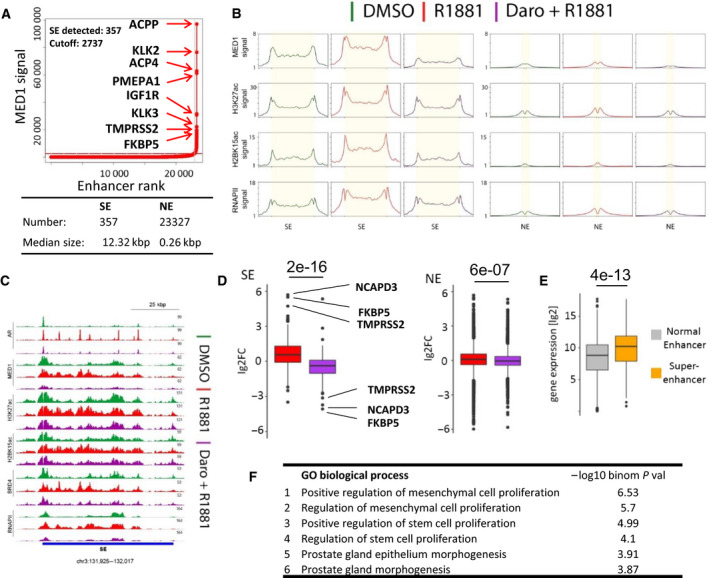
SE activation by R1881 and inhibition by darolutamide. (A) Overview of SEs identified in VCaP cells by ROSE and associated genes. The dotted red line shows the cutoff threshold for SEs. (B) Averaged protein occupancy and histone modification profiles at SEs (left) and NEs (right) for the treatments mentioned. (C) Top‐ranked SE identified in A is shown with indicated protein occupancies and histone modifications following the mentioned treatments. Bottom blue bar shows the SE region. (D) Log_2_ fold‐transformed gene expression changes (lg2FC) of SE‐ and NE‐associated genes after R1881 induction compared to DMSO (red), or after R1881 plus darolutamide compared to R1881 (purple). The top three R1881‐regulated genes are highlighted with their names. Numbers above the bar plots are the *t*‐test *P*‐values of the comparison between the groups. (E) Log_2_‐transformed expression values measured in R1881 condition of genes associated with NEs or SEs. The number above the bar plots is the *t*‐test *P*‐value of the comparison between the groups. (F) Molecular Signature Database gene sets significantly enriched with the genes proximal to SEs identified with GREAT and ranked by statistical significance. All sets are < 0.05 FDR value.

**Fig. 5:**
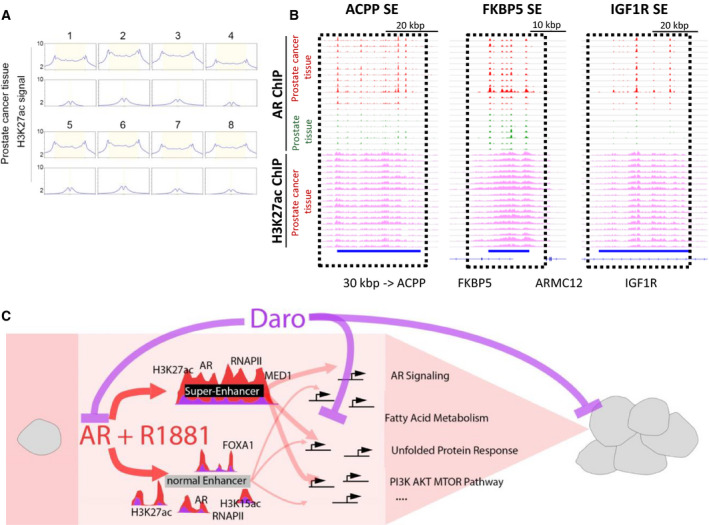
AR and H3K27ac are enriched at SEs in PCa tissue. (A) H3K27ac signal in four PCa tissues shown at SEs and NEs identified in Fig. 4A. Normalized intensity count is shown on the *y*‐axis. (B) AR ChIP‐seq signal at selected SEs identified in Fig. 4A in normal (green) and cancer (red) prostate tissues, together with H3K27ac signals from PCa tissues. AR and H3K27ac signals groups are scaled separately. Blue bars show the identified SE regions. (C) Molecular mechanistic model of the block of R1881‐mediated AR signaling leading to inhibition of NE and SE function, and transcription inhibition.

Super‐enhancers are major regulators of oncogene expression in different cancer types but the impact of AR antagonists on their function has not been explored yet. We used the ROSE algorithm based on the signal of MED1, a SE hallmark, to identify SEs at AR‐binding regions in R1881‐treated VCaP cells. We thereby identified 357 SEs with a median size of 12.3 kbp and 23 327 NEs with a median size of 0.26 kbp in VCaP cells (Fig. 4A). This was comparable to the number and size of MED1‐defined SEs found in other cell types (Loven *et al*., 2013; Whyte *et al*., 2013). SE‐associated genes were selected based on an overlap with the gene body or 50 kbp upstream of the TSS. Importantly, the genes included hallmark AR signaling genes such as KLK2, KLK3, TMPRSS2, and FKBP5 (Fig. 4A). Beside high binding of MED1, the SE sites had elevated H3K27ac and H2BK15ac levels, as well as higher RNA polymerase II (RNAPII) binding, compared to NEs (Fig. 4B). These marks increased upon R1881 treatment and were markedly reduced by additional darolutamide treatment, as shown globally and at highlighted SEs (Fig. 4B,C). Consistently, mRNA expression of SE‐associated genes was increased with R1881 treatment and inhibited by additional darolutamide treatment (Fig. 4D). SE‐associated genes were expressed at significantly higher levels compared to NE‐associated genes, as previously described in other cancer types (Fig. 4E). To identify the associated molecular pathways, we performed GREAT analysis of the SE regions and found a significant enrichment for R1881 response genes or ‘genes up‐regulated in PCa samples’ (Table S12), but interestingly also for ‘positive regulation of mesenchymal cell proliferation’, ‘positive regulation of stem cell proliferation’, and ‘prostate gland morphogenesis’, indicating that besides controlling AR hallmark genes, SEs fulfill other important functions (Fig. 4F, Table S12). Next, we looked at healthy and PCa tissue samples to determine whether the SE regions we identified in cell lines were also active in PCa patients. Publicly available AR ChIP‐seq data from healthy and PCa patients, and H3K27ac ChIP‐seq data from PCa patients were analyzed (Pomerantz *et al*., 2015). Indeed, we observed a broad enrichment of H3K27ac levels at SEs compared to NEs in PCa tissues, implying that the regions identified in VCaP cells were also active in PCa samples from different patients (Fig. 5A). Exemplarily, we analyzed three SE regions at the individual gene level and examined AR binding in healthy and tumor prostate tissues, and H3K27ac levels in PCa tissue (Fig. 5B). All three SEs showed dense clusters of high AR occupancy in PCa tissue with long stretches of H3K27ac covering the SE regions, similarly to the observation in VCaP cells.

Altogether these data indicate that SEs in PCa are in the vicinity of hallmark androgen response genes and probably have additional functions in regulating genes involved in cell proliferation and in stem cell properties. Importantly, darolutamide reduced the marked R1881‐mediated activation of SEs.

## 4 Discussion

In this work, we evaluated the molecular impact of the recently approved AR antagonist darolutamide in two different androgen‐dependent PCa cell lines and showed a potent and sustained reversal of genome‐wide AR binding and of the androgen‐induced transcriptome. A strong impact on AR signaling, fatty acid metabolism, unfolded protein response, and the PI3K/AKT/MTOR pathway was observed (Fig. 5C).

We performed a detailed transcriptomics analysis of VCaP and LAPC4 PCa cells treated for a comparatively short time to avoid a bias linked to growth conditions. We observed a strong impact of darolutamide on gene expression changes induced by androgen treatment. Overall, many more genes were regulated in VCaP cells, in line with the higher AR levels compared to LAPC4 cells (Sugawara *et al*., 2019). No comparable global transcriptome data are published for the anti‐androgens enzalutamide and apalutamide in these two cell lines, but previous analysis of a few selected genes indicates that these two compounds also inhibit androgen‐stimulated expression (Sugawara *et al*., 2019). To further elaborate on these findings on gene expression, we determined the global AR cistrome in the same two cell lines. More AR‐binding sites were detected in VCaP cells compared to LAPC4 cells, in line with the transcriptome data. VCaP cells also express high levels of AR‐V7, but this is not likely to contribute to additional binding peaks, as this splice variant binds to the same sites as the full‐length AR form (Cato *et al*., 2019). A detailed analysis of the AR cistrome allowed the identification of two main categories of AR‐binding regions, one smaller group with features of enhancer function and marked with FOXA1, BRD4, and H3K27ac (groups 1, 3, 5) and one larger group with initially low levels of these binding factors and modifications that were then increased upon R1881 treatment (groups 2, 4). AR binding to sites which gained transcriptional activity, as implied by H3K27ac, H3K18ac, and H2BK15ac, was blocked more strongly by darolutamide than AR binding to already established enhancers active in the DMSO‐only condition. As group 1 was also highly decorated by the AR in normal prostate tissue, it is possible that the AR‐binding sites it includes phenotypically define the prostate cell lineage. The most salient feature of AR sites already occupied in the DMSO condition was the presence of high levels of FOXA1 and BRD4, both of which interact with the AR and may foster DNA binding or AR recruitment to these sites (Gao *et al*., 2003; Lupien *et al*., 2008; Zhao *et al*., 2014). However, FOXA1/AR interaction alone is not sufficient for recruitment of AR as FOXA1 sites without AR occupancy exist (Lupien *et al*., 2008). BRD4 binds to acetylated histones to increase chromatin accessibility and forms a complex with the AR (Asangani *et al*., 2014). Additional members of this complex may further contribute to an increased binding of AR at specific genomic locations (Asangani *et al*., 2014).

Detailed studies on DNA motifs recognized by the AR highlight the important role of neighboring sequences and cooperating DNA‐binding factors to support AR binding (Toropainen *et al*., 2016; Wilson *et al*., 2016). In line with this, a set of reprogrammed AR‐binding regions characterized by FOXA1 and HOXB13 colocalization is acquired in prostate tumors (Copeland *et al*., 2019; Pomerantz *et al*., 2015). FOXA1 acts upstream of the AR (Zhao *et al*., 2016), and we show here that darolutamide decreased androgen‐stimulated FOXA1 occupancy, probably as a consequence of the blockade of AR binding. This supports the concept of a mutually cooperative binding of AR and FOXA1 at specific genomic regions (Gao *et al*., 2003; Lupien *et al*., 2008; Zhao *et al*., 2014). Very recently, mutations of FOXA1 that modify the chromatin landscape and ultimately disrupt epithelial differentiation have been described (Adams *et al*., 2019). These FOXA1 mutations define novel PCa subgroups, and it would be interesting to determine whether, compared to the wild‐type form, their chromatin interaction is similarly affected by an AR antagonist.

Super‐enhancers exert a major role in the progression of various tumor types including PCa (Baumgart *et al*., 2019; He *et al*., 2019; Zuber *et al*., 2017) and also contribute to resistance mechanisms involving SE‐binding factors such as BRD4 or FOXP1 (Bao *et al*., 2019; Chen *et al*., 2019; Natsume *et al*., 2019). SE regions have been identified in PCa models but the associated genes and the impact of AR antagonists have not been dissected. Here, we show that SEs were present in the vicinity of several hallmark AR signaling genes, including the pharmacodynamic biomarker KLK3, and that darolutamide strongly reduced their activation. SEs are part of dynamic, phase‐separated condensates that concentrate the transcriptional apparatus (Hnisz *et al*., 2017; Sabari *et al*., 2018). They are particularly sensitive to inhibition of their components in line with the strong effect observed for darolutamide on gene regulation (Moilanen *et al*., 2015; Sugawara *et al*., 2019). Aberrantly activated SEs have been described in different tumor types, and further studies will give more insights into the role of SEs in PCa.

## 5 Conclusion

In this work, we showed for the first time the genome‐wide effects of the novel AR antagonist darolutamide on the AR cistrome and transcriptome in PCa models. The strong reduction of AR binding to the genome elicited by darolutamide blocked NE and SE activation, which down‐tuned several downstream pathways important for PCa proliferation. We identified genomic regions with different affinities for the AR under different treatment conditions. Regions of group 1 have robust AR occupancy in cellular models, and healthy and diseased prostate tissue and can be considered as *bona fide* AR‐bound regions in the human genome. Importantly, different enrichments for FOXA1, BRD4, and H3K27ac were observed in the AR‐binding groups defined so that varying responses to compounds that address these factors can be expected. These factors may form phase‐separated condensates at specific regulatory regions, as recently described for DNA‐bound OCT4/Med1 (Shrinivas *et al*., 2019), and the role of the internally disorder region of the AR N‐terminal domain (McEwan, 2012) in local interactions should be further explored. Our data on SEs bound by the AR expand on the role of SEs recently shown for other cancer types, in some cases allowing for stratification predictive of treatment response (Cejas *et al*., 2019; Gelato *et al*., 2018; Zhang *et al*., 2016). Interestingly, in breast cancer, estrogen receptor‐bound SEs are also bound by MED1 and FOXA1, which act as facilitators for the interaction with neighboring enhancers (Bojcsuk *et al*., 2017), and lists of highly active SEs which potentially play a critical role in this tumor type have just been reported (Hazan *et al*., 2019; Li *et al*., 2019). Another important recent finding is that oncogenic SEs are inclined to undergo double‐strand breaks and are therefore highly vulnerable to deficiencies in cellular DNA repair mechanisms. This suggests that combining endocrine therapy with agents addressing DNA damage repair will have increased antitumor efficacy and corresponding clinical studies have been initiated in breast cancer patients (Plummer *et al*., 2018). Concerning PCa, cotreatments showing superior antitumor efficacy in preclinical xenograft models include the combination of enzalutamide with the PARP inhibitor olaparib (Li *et al*., 2017) and the combination of darolutamide with the ATR inhibitor BAY 1895344 (Wengner *et al*., 2020). This gives hope that novel treatment strategies aiming at blocking SEs from different angles will offer new therapy options to patients in the near future.

## Conflict of interest

The authors are employees and/or own shares of Bayer AG.

## Author contributions

SJB, RL, DM, and BH were involved in the study design. SJB conducted all the experimental parts. SJB and EN performed the bioinformatic analyses. RL carried out the sequencing experiments. RN performed the statistical analyses. SJB wrote the manuscript, and EN and BH made the main changes. All authors read and approved the final manuscript.

## Supporting information


**Fig. S1**. Gene Set Enrichment Analysis of the transcriptional impact of darolutamide compared to the R1881 stimulation in VCaP and LAPC4 cells.
**Fig. S2** **.** (A) Heatmaps showing signals of single AR ChIP‐seq replicates centered at R1881‐induced AR‐binding sites including a 2.5 kb region up‐ and downstream. (B) Overlap of the defined AR‐binding groups in LAPC4 and VCaP cells sorted by the ratio given in percentage of overlapping regions relative to all regions in the respective LAPC4 group. (C) Motif analysis of AR‐binding clusters shown in a word cloud.
**Fig. S3** **.** AR ChIP‐seq signals in healthy prostate and PCa tissue samples shown with heatmaps and centered around the AR groups identified in VCaP cells.
**Fig. S4** **.** (A) ChIP‐seq signals of replicates for the DMSO, R1881 and darolutamide plus R1881 conditions centered at AR‐binding regions plus 2.5 kb up and downstream for AR groups identified in VCaP cells. (B) Heatmaps of ChIP‐seq signals from LAPC4 cells with averaged profiles for DMSO‐, R1881‐ and R1881‐ plus darolutamide‐treated samples at genomic regions bound by the AR after R1881 induction. (C) Heatmaps of ChIP‐seq signals from VCaP cells with averaged profiles for DMSO‐, R1881‐ and R1881‐ plus darolutamide‐treated samples at genomic regions bound by the AR after R1881 induction.
**Fig. S5** **.** (A) Average signal plots of FOXA1 ChIP‐seq data in the identified LAPC4 cell groups. (B) BRD4 ChIP‐seq signals in VCaP cells at defined AR‐binding groups
**Table S1** **.** List of antibodies used in ChIP‐seq experiments.
**Table S2** **.** GSEA output of hallmark gene sets enriched among the genes differentially expressed between darolutamide 2 µm + 1 nm R1881‐treated VCaP vs 1 nm R1881‐treated samples at 22 h post treatment.
**Table S3** **.** GSEA output of hallmark gene sets enriched among the genes differentially expressed between darolutamide 2 µm + 1 nm R1881‐treated VCaP vs 1 nm R1881‐treated samples 8 h post treatment.
**Table S4** **.** GSEA output of hallmark gene sets enriched among the genes differentially expressed between darolutamide 500 nm + 1 nm R1881‐treated VCaP vs 1 nm R1881‐treated samples 22 h post treatment.
**Table S5** **.** GSEA output of hallmark gene sets enriched among the genes differentially expressed between darolutamide 500 nm + 1 nm R1881‐treated VCaP vs 1 nm R1881‐treated samples 8 h post treatment.
**Table S6** **.** GSEA output of hallmark gene sets enriched among the genes differentially expressed between darolutamide 2 µm + 1 nm R1881‐treated LAPC4 vs 1 nm R1881‐treated samples 22 h post treatment.
**Table S7** **.** GSEA output of hallmark gene sets enriched among the genes differentially expressed between darolutamide 2 µm + 1 nm R1881‐treated LAPC4 vs 1 nm R1881‐treated samples 8 h post treatment.
**Table S8** **.** GSEA output of hallmark gene sets enriched among the genes differentially expressed between darolutamide 500 nm + 1 nm R1881‐treated LAPC4 vs 1 nm R1881‐treated samples 22 h post treatment.
**Table S9** **.** GSEA output of hallmark gene sets enriched among the genes differentially expressed between darolutamide 500 nm + 1 nm R1881‐treated LAPC4 vs 1 nm R1881‐treated samples 8 h post treatment.
**Table S10** **.** Location of constitutive AR‐binding sites found under all treatment conditions in VCaP and LAPC4 cells and corresponding genes identified based on AR peak location within 20 kbp upstream of the TSS or in the gene body.
**Table S11** **.** Analysis of genes proximal to the AR‐binding regions identified in VCaP and LAPC4 cells by GREAT analysis.
**Table S12** **.** MSigDB sets of genes associated with SEs in VCaP cells analyzed by GREAT.
