# Supplementary material for: Darolutamide antagonizes androgen signaling by blocking enhancer and super‐enhancer activation
Source: Mol Oncol. 2020 Jun 5;14(9):2022–39. doi: 10.1002/1878-0261.12693 (PMC7463324; doi:10.1002/1878-0261.12693)
Supplement: Supplementary file 1 — Fig. S1. Gene Set Enrichment Analysis of the transcriptional impact of darolutamide compared to the R1881 stimulation in VCaP and LAPC4 cells. Fig. S2 . (A) Heatmaps showing signals of single AR ChIP‐seq replicates centered at R1881‐induced AR‐binding sites including a 2.5 kb region up‐ and downstream. (B) Overlap of the defined AR‐binding groups in LAPC4 and VCaP cells sorted by the ratio given in percentage of overlapping regions relative to all regions in the respective LAPC4 group. (C) Motif analysis of AR‐binding clusters shown in a word cloud. Fig. S3 . AR ChIP‐seq signals in healthy prostate and PCa tissue samples shown with heatmaps and centered around the AR groups identified in VCaP cells. Fig. S4 . (A) ChIP‐seq signals of replicates for the DMSO, R1881 and darolutamide plus R1881 conditions centered at AR‐binding regions plus 2.5 kb up and downstream for AR groups identified in VCaP cells. (B) Heatmaps of ChIP‐seq signals from LAPC4 cells with averaged profiles for DMSO‐, R1881‐ and R1881‐ plus darolutamide‐treated samples at genomic regions bound by the AR after R1881 induction. (C) Heatmaps of ChIP‐seq signals from VCaP cells with averaged profiles for DMSO‐, R1881‐ and R1881‐ plus darolutamide‐treated samples at genomic regions bound by the AR after R1881 induction. Fig. S5 . (A) Average signal plots of FOXA1 ChIP‐seq data in the identified LAPC4 cell groups. (B) BRD4 ChIP‐seq signals in VCaP cells at defined AR‐binding groups Table S1 . List of antibodies used in ChIP‐seq experiments. Table S2 . GSEA output of hallmark gene sets enriched among the genes differentially expressed between darolutamide 2 µm + 1 nm R1881‐treated VCaP vs 1 nm R1881‐treated samples at 22 h post treatment. Table S3 . GSEA output of hallmark gene sets enriched among the genes differentially expressed between darolutamide 2 µm + 1 nm R1881‐treated VCaP vs 1 nm R1881‐treated samples 8 h post treatment. Table S4 . GSEA output of hallmark gene sets enriched among the genes differe [file MOL2-14-2022-s001.pdf]

Supplementary Files
Figure Legends

Fig. S1. Gene Set Enrichment Analysis of the transcriptional impact of darolutamide compared to the R1881 stimulation at the mentioned time points and concentrations in VCaP and LAPC4 cells. Hallmark gene sets were selected as mentioned in Fig 1.

Fig. S2. (A) Heatmaps showing signals of single AR ChIP-seq replicates centered at R1881-induced AR-binding sites including a 2.5 kb region up- and downstream. The signals are arranged in a descending order based on the R1881 sample signal. (B) Overlap of the defined AR-binding groups in LAPC4 and VCaP cells sorted by the ratio given in percentage of overlapping regions relative to all regions in the respective LAPC4 group. (C) Motif analysis of the AR-binding clusters identified in Fig. 2B shown in a word cloud for the VCaP and LAPC4 cells in the upper and lower panels, respectively. Word size corresponds to absolute z-scores of the motif enrichment. Motif colors correspond to DNA-binding domain families.

Fig. S3. AR ChIP-seq signals in healthy prostate and PCa tissue samples are shown with heatmaps and centered around the AR groups identified in VCaP cells (Fig. 2). The green and red boxes highlight healthy and PCa tissue samples, respectively. The AR occupancy intensity scale is shown on the right hand side beside each heatmap and scaled uniformly between all samples. Data are taken from GSE96652.

Fig. S4. (A) ChIP-seq signals of replicates for the DMSO, R1881 and darolutamide plus R1881 conditions centered at AR-binding regions plus 2.5 kb up and downstream for AR groups identified in VCaP cells. Regions are sorted in descending order based on data from the R1881-treated samples. (B) Heatmaps of ChIP-seq signals from LAPC4 cells with averaged profiles for DMSO-, R1881- and R1881- plus darolutamide-treated samples at genomic regions bound by the AR after R1881 induction. Regions are sorted to the R1881 condition in descending AR-binding intensity. (C) Heatmaps of ChIP-seq signals from VCaP cells with averaged profiles for DMSO-, R1881- and R1881- plus darolutamide-treated samples at genomic regions bound by the AR after R1881 induction. Regions are sorted to the R1881 condition in descending AR-binding intensity.

Fig. S5. (A) Average signal plots of FOXA1 ChIP-seq data in the identified LAPC4 cell groups. ChIP-seq data were retrieved from GSE123625. (B) BRD4 ChIP-seq signals in VCaP cells at the AR-binding groups defined in Fig. 2A are shown after treatment with DMSO or dihydrotestosterone (DHT). Samples were retrieved from GSE55062.

Supplementary Files
Table Legends

Table S1. List of antibodies used in ChIP-seq experiments.

Table S2. GSEA output of hallmark gene sets enriched among the genes differentially expressed between darolutamide 2 µM + 1 nM R1881-treated VCaP vs 1 nM R1881-treated samples at 22 h post treatment.

Table S3 GSEA output of hallmark gene sets enriched among the genes differentially expressed between darolutamide 2 µM + 1 nM R1881-treated VCaP vs 1 nM R1881-treated samples 8 h post treatment.

Table S4. GSEA output of hallmark gene sets enriched among the genes differentially expressed between darolutamide 500 nM + 1 nM R1881-treated VCaP vs 1 nM R1881-treated samples 22 h post treatment.

Table S5. GSEA output of hallmark gene sets enriched among the genes differentially expressed between darolutamide 500 nM + 1 nM R1881-treated VCaP vs 1 nM R1881-treated samples 8 h post treatment.

Table S6. GSEA output of hallmark gene sets enriched among the genes differentially expressed between darolutamide 2 µM + 1 nM R1881-treated LAPC4 vs 1 nM R1881-treated samples 22 h post treatment.

Table S7. GSEA output of hallmark gene sets enriched among the genes differentially expressed between darolutamide 2 µM + 1 nM R1881-treated LAPC4 vs 1 nM R1881-treated samples 8 h post treatment.

Table S8. GSEA output of hallmark gene sets enriched among the genes differentially expressed between darolutamide 500 nM + 1 nM R1881-treated LAPC4 vs 1 nM R1881-treated samples 22 h post treatment.

Table S9. GSEA output of hallmark gene sets enriched among the genes differentially expressed between darolutamide 500 nM + 1 nM R1881-treated LAPC4 vs 1 nM R1881-treated samples 8 h post treatment.

Table S10. Location of constitutive AR-binding sites found under all treatment conditions in VCaP and LAPC4 cells and corresponding genes identified based on AR peak location within 20 kbp upstream of the TSS or in the gene body (See also Fig. S2B).

Table S11. Analysis of genes proximal to the AR-binding regions identified in VCaP and LAPC4 cells by GREAT analysis. Significantly enriched gene sets with a FDR < 0.05 are shown and ordered by log10(binominal p-val.). The upper part shows enriched sets form VCaP cells and the lower panel enriched sets from LAPC4 cells.

Table S12. MSigDB sets of genes associated with SEs in VCaP cells analyzed by GREAT. Statistical enrichment scores are shown.

Suppl. Fig. S1

VCaP

08 h 2 µM darolutamide + 1 nM R1881

Androgen Response

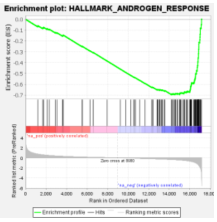

Normalized Enrichment Score (NES)	-2.60
Nominal p-value	0
FDR q-value	0

Fatty Acid Metabolism

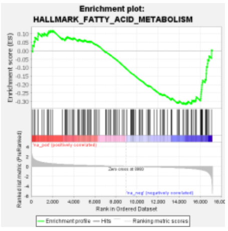

Normalized Enrichment Score (NES)	-1.21
Nominal p-value	0.12
FDR q-value	0.22

PI3K AKT mTOR Signaling

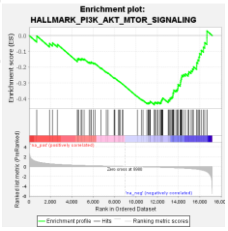

Normalized Enrichment Score (NES)	-1.60
Nominal p-value	0.01
FDR q-value	0.03

08 h 500 nM darolutamide + 1 nM R1881

Androgen Response

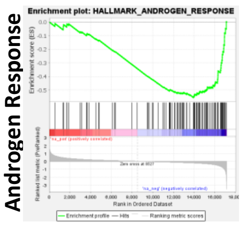

Normalized Enrichment Score (NES)	-2.08
Nominal p-value	0
FDR q-value	0

Fatty Acid Metabolism

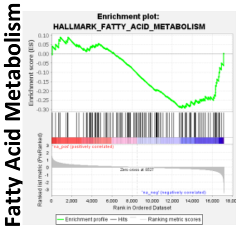

Normalized Enrichment Score (NES)	-1.15
Nominal p-value	0.19
FDR q-value	0.31

PI3K AKT mTOR Signaling

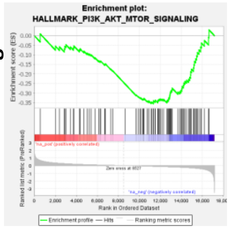

Normalized Enrichment Score (NES)	-1.31
Nominal p-value	0.07
FDR q-value	0.12

22 h 500 nM darolutamide + 1 nM R1881

Androgen Response

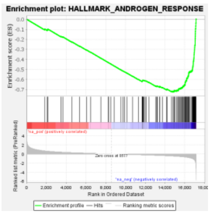

Normalized Enrichment Score (NES)	-3.04
Nominal p-value	0
FDR q-value	0

Fatty Acid Metabolism

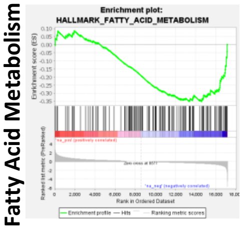

Normalized Enrichment Score (NES)	-1.57
Nominal p-value	0
FDR q-value	0.03

PI3K AKT mTOR Signaling

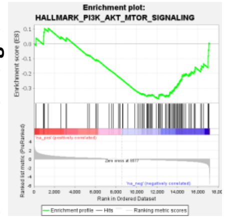

Normalized Enrichment Score (NES)	-1.53
Nominal p-value	0
FDR q-value	0.03

LAPC4

08 h 2 µM darolutamide + 1 nM R1881

Androgen Response

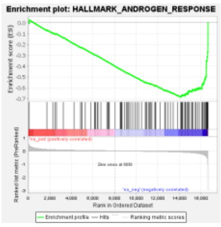

Normalized Enrichment Score (NES)	-2.31
Nominal p-value	0
FDR q-value	0

Fatty Acid Metabolism

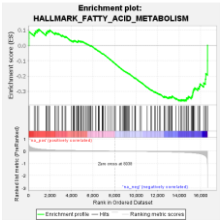

Normalized Enrichment Score (NES)	1.27
Nominal p-value	0.07
FDR q-value	0.40

08 h 500 nM darolutamide + 1 nM R1881

Androgen Response

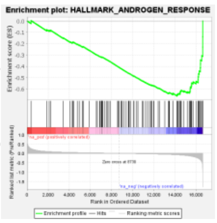

Normalized Enrichment Score (NES)	-2.17
Nominal p-value	0
FDR q-value	0

Fatty Acid Metabolism

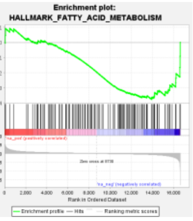

Normalized Enrichment Score (NES)	-1.29
Nominal p-value	0.06
FDR q-value	0.57

22 h 500 nM darolutamide + 1 nM R1881

Androgen Response

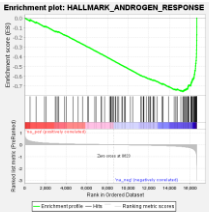

Normalized Enrichment Score (NES)	-2.69
Nominal p-value	0
FDR q-value	0

Fatty Acid Metabolism

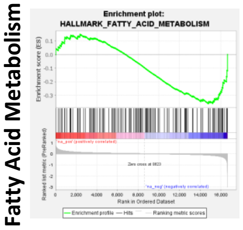

Normalized Enrichment Score (NES)	-1.35
Nominal p-value	0.036
FDR q-value	0.14

Suppl. Fig. S2

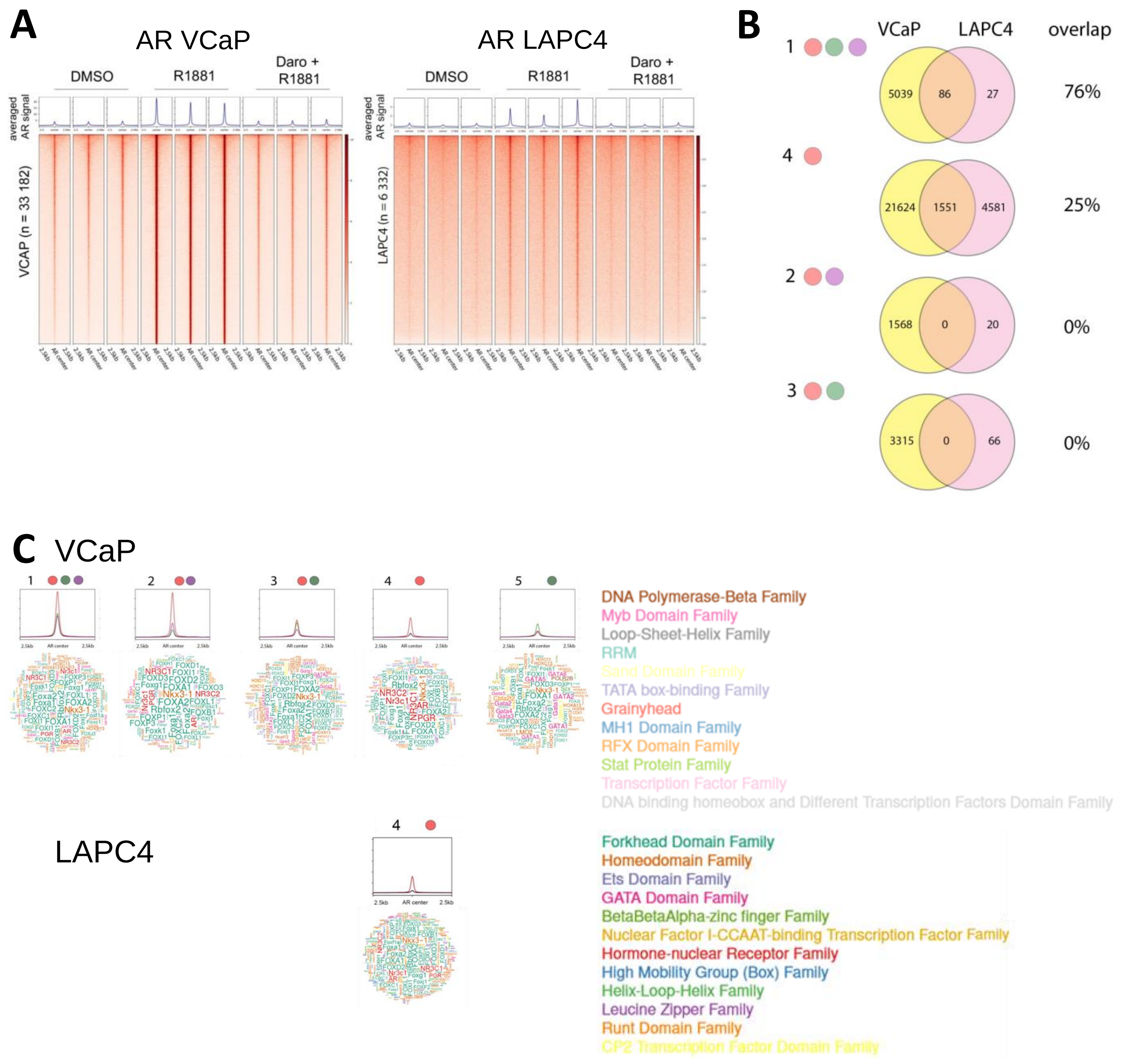
A
AR VCaP
AR LAPC4
DMSO
R1881
Daro + R1881
averaged AR signal
VCAP (n = 33 182)
LAPC4 (n = 6 332)
B
VCaP
LAPC4
overlap
1
5039
86
27
76%
4
21624
1551
4581
25%
2
1568
0
20
0%
3
3315
0
66
0%
C
VCaP
LAPC4
DNA Polymerase-Beta Family
Myb Domain Family
Loop-Sheet-Helix Family
RRM
Sand Domain Family
TATA box-binding Family
Grainyhead
MH1 Domain Family
RFX Domain Family
Stat Protein Family
Transcription Factor Family
DNA binding homeobox and Different Transcription Factors Domain Family
Forkhead Domain Family
Homeodomain Family
Ets Domain Family
GATA Domain Family
BetaBetaAlpha-zinc finger Family
Nuclear Factor I-CCAAT-binding Transcription Factor Family
Hormone-nuclear Receptor Family
High Mobility Group (Box) Family
Helix-Loop-Helix Family
Leucine Zipper Family
Runt Domain Family
CP2 Transcription Factor Domain Family

Suppl. Fig. S3

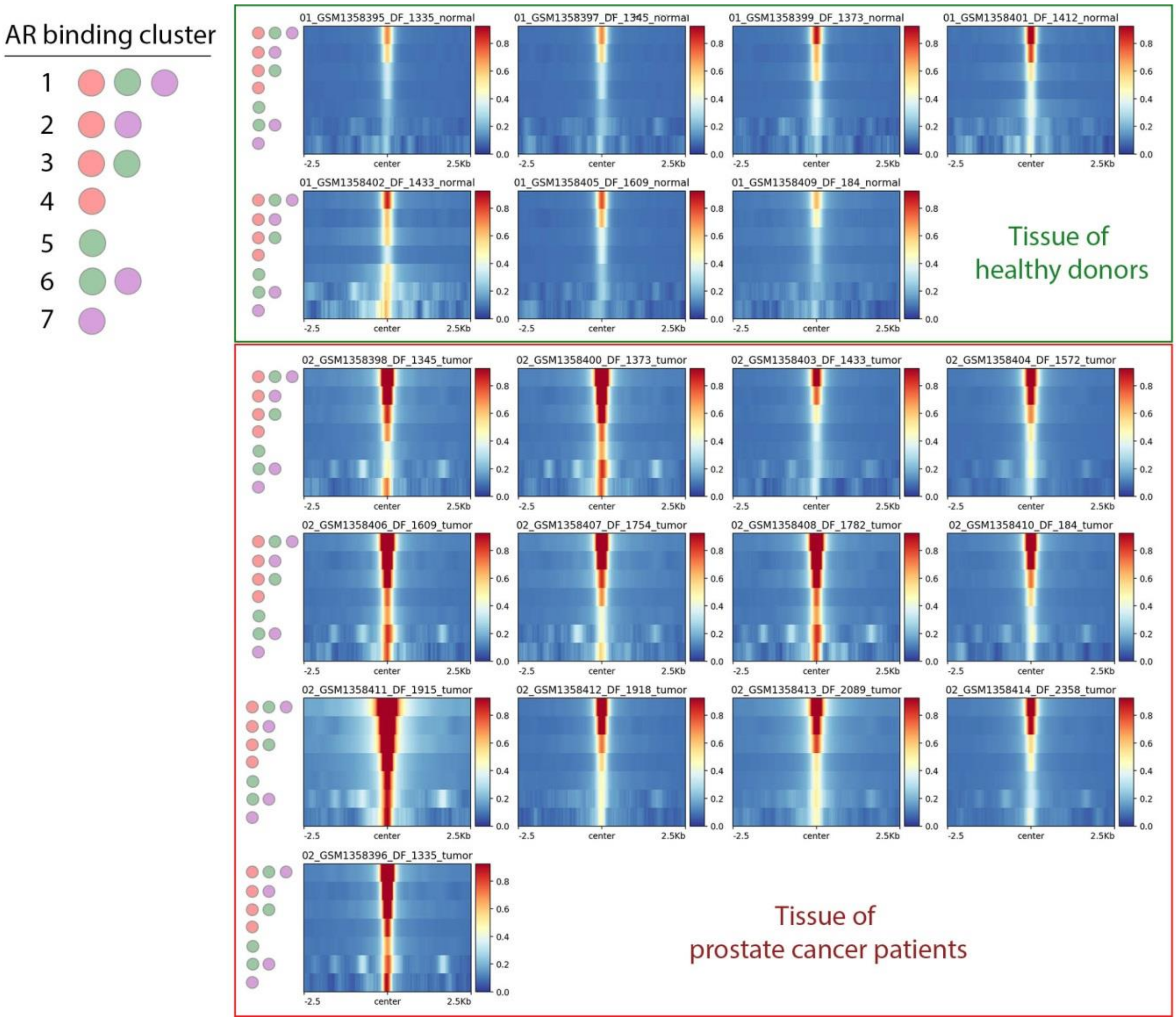
AR binding cluster
1
2
3
4
5
6
7
01_GSM1358395_DF_1335_normal
01_GSM1358397_DF_1345_normal
01_GSM1358399_DF_1373_normal
01_GSM1358401_DF_1412_normal
01_GSM1358402_DF_1433_normal
01_GSM1358405_DF_1609_normal
01_GSM1358409_DF_184_normal
Tissue of healthy donors
02_GSM1358398_DF_1345_tumor
02_GSM1358400_DF_1373_tumor
02_GSM1358403_DF_1433_tumor
02_GSM1358404_DF_1572_tumor
02_GSM1358406_DF_1609_tumor
02_GSM1358407_DF_1754_tumor
02_GSM1358408_DF_1782_tumor
02_GSM1358410_DF_184_tumor
02_GSM1358411_DF_1915_tumor
02_GSM1358412_DF_1918_tumor
02_GSM1358413_DF_2089_tumor
02_GSM1358414_DF_2358_tumor
02_GSM1358396_DF_1335_tumor
Tissue of prostate cancer patients
-2.5
center
2.5Kb
0.0
0.2
0.4
0.6
0.8

Suppl. Fig. S4

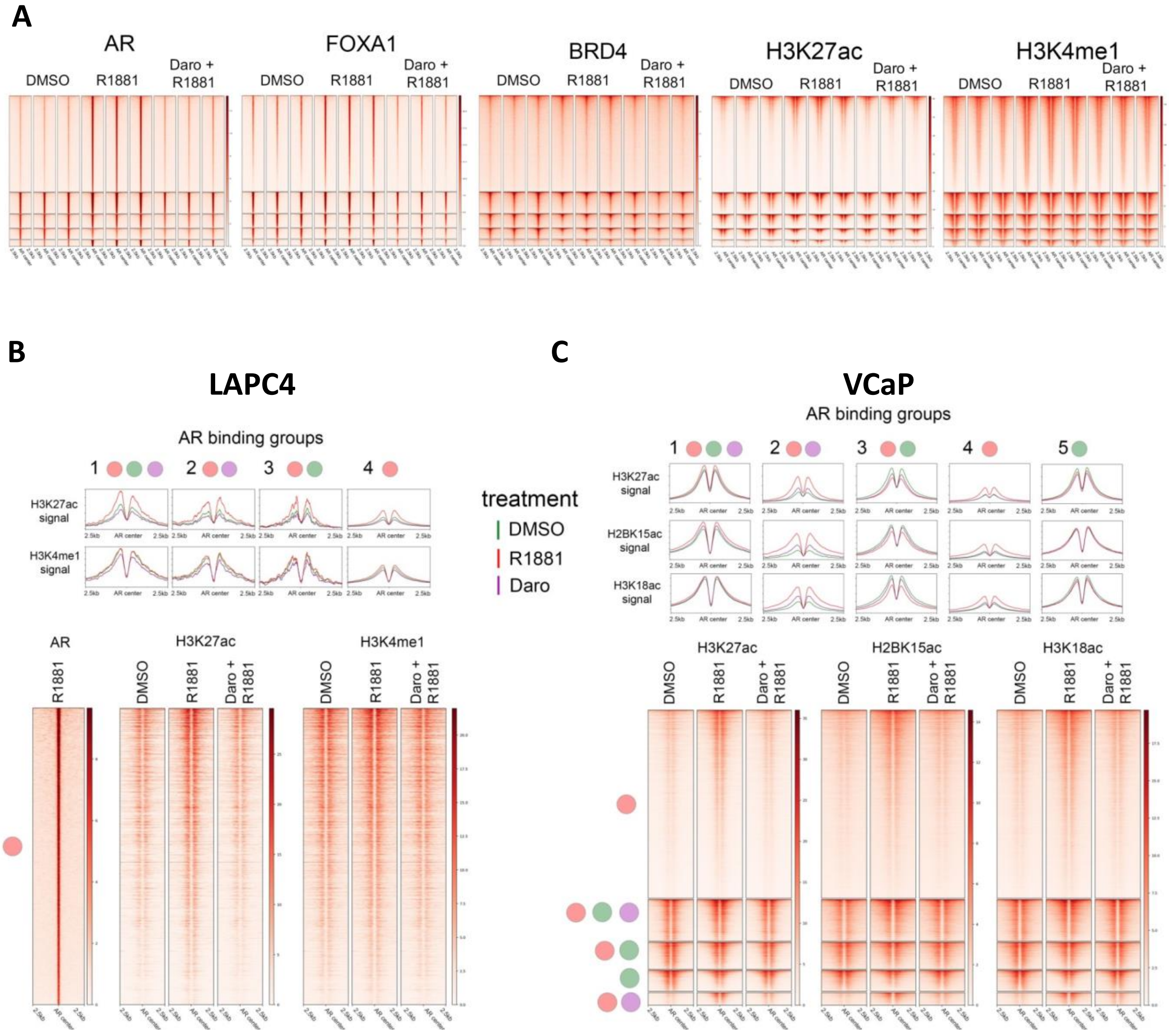
A
AR
FOXA1
BRD4
H3K27ac
H3K4me1
DMSO
R1881
Daro + R1881
B
LAPC4
AR binding groups
H3K27ac signal
H3K4me1 signal
2.5kb
AR center
treatment
DMSO
R1881
Daro
AR
H3K27ac
H3K4me1
C
VCaP
AR binding groups
H3K27ac signal
H2BK15ac signal
H3K18ac signal
H3K27ac
H2BK15ac
H3K18ac

Suppl. Fig. S5

A LAPC4

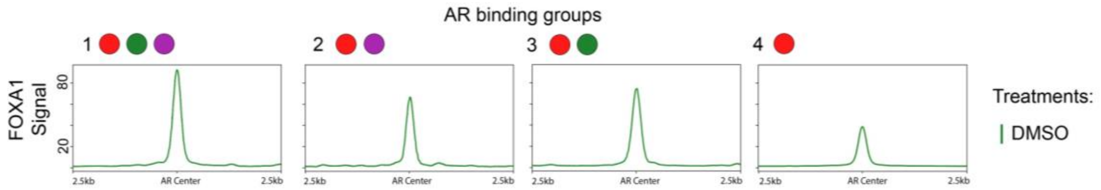

B VCaP

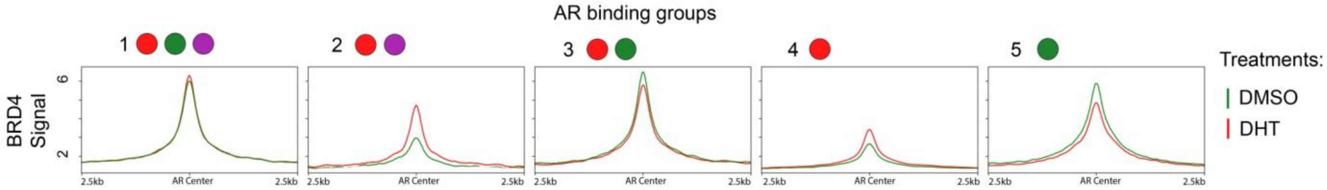

Suppl. Table S1

Target protein	Vendor	Catalog number
AR	Abcam	ab74272
BRD4	Bethyl Laboratories	A301-985A100
FOXA1	Abcam	ab23738
MED1	Bethyl Laboratories	A300-793A
RNAPII	Abcam	ab26721
H3K27ac	Diagenode	C15410196
H3K4me1	Diagenode	C15410194
H3K18ac	Diagenode	C15410139
H2BK15ac	Diagenode	C15410220

Suppl. Table S2

VCaP.daroHigh.R1881.22h

DOWN-regulated pathways

NAME	ES	NES	NOM p-val	FDR q-val
HALLMARK_ANDROGEN_RESPONSE	-0.76	-3.12	0.00	0.00
HALLMARK_PROTEIN_SECRETION	-0.50	-2.08	0.00	0.00
HALLMARK_PI3K_AKT_MTOR_SIGNALING	-0.45	-1.82	0.00	0.00
HALLMARK_HYPOXIA	-0.40	-1.76	0.00	0.01
HALLMARK_CHOLESTEROL_HOMEOSTASIS	-0.45	-1.74	0.00	0.01
HALLMARK_TNFA_SIGNALING_VIA_NFKB	-0.37	-1.61	0.00	0.02
HALLMARK_FATTY_ACID_METABOLISM	-0.37	-1.60	0.00	0.02
HALLMARK_ESTROGEN_RESPONSE_LATE	-0.36	-1.59	0.00	0.02
HALLMARK_UNFOLDED_PROTEIN_RESPONSE	-0.37	-1.57	0.00	0.02
HALLMARK_ESTROGEN_RESPONSE_EARLY	-0.33	-1.50	0.01	0.03
HALLMARK_MTORC1_SIGNALING	-0.33	-1.49	0.00	0.03
HALLMARK_BILE_ACID_METABOLISM	-0.35	-1.41	0.03	0.06
HALLMARK_ADIPOGENESIS	-0.29	-1.33	0.01	0.10
HALLMARK_HEME_METABOLISM	-0.29	-1.31	0.02	0.10
HALLMARK_TGF_BETA_SIGNALING	-0.36	-1.30	0.09	0.10
HALLMARK_IL2_STAT5_SIGNALING	-0.29	-1.27	0.05	0.12
HALLMARK_P53_PATHWAY	-0.26	-1.18	0.09	0.21
HALLMARK_COAGULATION	-0.30	-1.18	0.18	0.20

UP-regulated pathways

NAME	ES	NES	NOM p-val	FDR q-val
HALLMARK_E2F_TARGETS	0.64	2.57	0.00	0.00
HALLMARK_G2M_CHECKPOINT	0.52	2.11	0.00	0.00
HALLMARK_EPITHELIAL_MESENCHYMAL_TRANSITION	0.54	2.08	0.00	0.00
HALLMARK_MYC_TARGETS_V2	0.61	2.08	0.00	0.00
HALLMARK_MYC_TARGETS_V1	0.46	1.88	0.00	0.00
HALLMARK_KRAS_SIGNALING_DN	0.43	1.56	0.00	0.03
HALLMARK_HEDGEHOG_SIGNALING	0.52	1.54	0.04	0.04
HALLMARK_KRAS_SIGNALING_UP	0.40	1.53	0.01	0.04
HALLMARK_ANGIOGENESIS	0.52	1.47	0.06	0.05
HALLMARK_WNT_BETA_CATENIN_SIGNALING	0.47	1.44	0.04	0.06
HALLMARK_INFLAMMATORY_RESPONSE	0.36	1.33	0.07	0.16
HALLMARK_INTERFERON_ALPHA_RESPONSE	0.37	1.31	0.08	0.16

Suppl. Table S3

VCaP.daroHigh.R1881.08h

UP-regulated pathways

NAME	ES	NES	NOM p-val	FDR q-val
HALLMARK_MYC_TARGETS_V2	0.52	1.72	0.00	0.02
HALLMARK_INTERFERON_ALPHA_RESPONSE	0.51	1.71	0.00	0.01
HALLMARK_KRAS_SIGNALING_UP	0.40	1.47	0.01	0.12
HALLMARK_INTERFERON_GAMMA_RESPONSE	0.38	1.42	0.02	0.15

DOWN-regulated pathways

NAME	ES	NES	NOM p-val	FDR q-val
HALLMARK_ANDROGEN_RESPONSE	-0.70	-2.59	0.00	0.00
HALLMARK_UNFOLDED_PROTEIN_RESPONSE	-0.53	-1.98	0.00	0.00
HALLMARK_PROTEIN_SECRETION	-0.53	-1.93	0.00	0.00
HALLMARK_HYPOXIA	-0.47	-1.85	0.00	0.00
HALLMARK_CHOLESTEROL_HOMEOSTASIS	-0.50	-1.70	0.00	0.01
HALLMARK_PI3K_AKT_MTOR_SIGNALING	-0.44	-1.60	0.00	0.02
HALLMARK_UV_RESPONSE_UP	-0.41	-1.56	0.01	0.03
HALLMARK_MTORC1_SIGNALING	-0.37	-1.47	0.00	0.06
HALLMARK_TNFA_SIGNALING_VIA_NFKB	-0.37	-1.44	0.01	0.07
HALLMARK_MITOTIC_SPINDLE	-0.35	-1.43	0.00	0.07
HALLMARK_APICAL_JUNCTION	-0.35	-1.37	0.02	0.11
HALLMARK_GLYCOLYSIS	-0.34	-1.35	0.01	0.11
HALLMARK_ESTROGEN_RESPONSE_LATE	-0.34	-1.33	0.03	0.12
HALLMARK_ESTROGEN_RESPONSE_EARLY	-0.32	-1.29	0.06	0.15
HALLMARK_APOPTOSIS	-0.34	-1.28	0.09	0.16
HALLMARK_OXIDATIVE_PHOSPHORYLATION	-0.31	-1.25	0.07	0.17

Suppl. Table S4

VCaP.daroLow.R1881.22h

UP-regulated pathways

NAME	ES	NES	NOM p-val	FDR q-val
HALLMARK_E2F_TARGETS	0.71	2.86	0.00	0.00
HALLMARK_G2M_CHECKPOINT	0.61	2.47	0.00	0.00
HALLMARK_MYC_TARGETS_V2	0.65	2.20	0.00	0.00
HALLMARK_EPITHELIAL_MESENCHYMAL_TRANSITION	0.55	2.11	0.00	0.00
HALLMARK_MYC_TARGETS_V1	0.51	2.05	0.00	0.00
HALLMARK_KRAS_SIGNALING_DN	0.44	1.60	0.00	0.02
HALLMARK_ANGIOGENESIS	0.57	1.59	0.02	0.02
HALLMARK_WNT_BETA_CATENIN_SIGNALING	0.47	1.44	0.05	0.09
HALLMARK_KRAS_SIGNALING_UP	0.38	1.41	0.03	0.10
HALLMARK_HEDGEHOG_SIGNALING	0.45	1.36	0.11	0.14
HALLMARK_INTERFERON_ALPHA_RESPONSE	0.39	1.35	0.08	0.13
HALLMARK_DNA_REPAIR	0.33	1.30	0.06	0.19
HALLMARK_MITOTIC_SPINDLE	0.32	1.29	0.05	0.18

DOWN-regulated pathways

NAME	ES	NES	NOM p-val	FDR q-val
HALLMARK_ANDROGEN_RESPONSE	-0.73	-3.04	0.00	0.00
HALLMARK_PROTEIN_SECRETION	-0.51	-2.17	0.00	0.00
HALLMARK_TNFA_SIGNALING_VIA_NFKB	-0.37	-1.69	0.00	0.02
HALLMARK_HYPOXIA	-0.37	-1.69	0.00	0.02
HALLMARK_CHOLESTEROL_HOMEOSTASIS	-0.42	-1.67	0.00	0.02
HALLMARK_FATTY_ACID_METABOLISM	-0.35	-1.57	0.00	0.03
HALLMARK_PI3K_AKT_MTOR_SIGNALING	-0.37	-1.53	0.01	0.03
HALLMARK_UNFOLDED_PROTEIN_RESPONSE	-0.34	-1.50	0.01	0.04
HALLMARK_HEME_METABOLISM	-0.32	-1.45	0.00	0.05
HALLMARK_BILE_ACID_METABOLISM	-0.35	-1.43	0.02	0.05
HALLMARK_ESTROGEN_RESPONSE_LATE	-0.30	-1.37	0.01	0.07
HALLMARK_ESTROGEN_RESPONSE_EARLY	-0.30	-1.36	0.01	0.07
HALLMARK_TGF_BETA_SIGNALING	-0.36	-1.32	0.06	0.09
HALLMARK_OXIDATIVE_PHOSPHORYLATION	-0.27	-1.24	0.03	0.14
HALLMARK_ADIPOGENESIS	-0.27	-1.23	0.07	0.15
HALLMARK_XENOBIOTIC_METABOLISM	-0.27	-1.19	0.08	0.18
HALLMARK_IL2_STAT5_SIGNALING	-0.26	-1.17	0.14	0.20

Suppl. Table S5

VCaP.daroLow.R1881.08h

UP-regulated pathways

NAME	ES	NES	NOM p-val	FDR q-val
HALLMARK_INTERFERON_ALPHA_RESPONSE	0.51	1.79	0.00	0.01
HALLMARK_KRAS_SIGNALING_UP	0.46	1.73	0.00	0.01
HALLMARK_INTERFERON_GAMMA_RESPONSE	0.41	1.60	0.00	0.03
HALLMARK_INFLAMMATORY_RESPONSE	0.40	1.48	0.01	0.08
HALLMARK_NOTCH_SIGNALING	0.46	1.38	0.09	0.17
HALLMARK_ALLOGRAFT_REJECTION	0.38	1.38	0.05	0.14
HALLMARK_EPITHELIAL_MESENCHYMAL_TRANSITION	0.36	1.37	0.03	0.13
HALLMARK_IL6_JAK_STAT3_SIGNALING	0.42	1.34	0.10	0.14
HALLMARK_APICAL_SURFACE	0.45	1.30	0.13	0.17
HALLMARK_E2F_TARGETS	0.31	1.27	0.04	0.19

DOWN-regulated pathways

NAME	ES	NES	NOM p-val	FDR q-val
HALLMARK_UNFOLDED_PROTEIN_RESPONSE	-0.58	-2.21	0.00	0.00
HALLMARK_ANDROGEN_RESPONSE	-0.56	-2.08	0.00	0.00
HALLMARK_PROTEIN_SECRETION	-0.47	-1.77	0.00	0.01
HALLMARK_HYPOXIA	-0.44	-1.74	0.00	0.01
HALLMARK_UV_RESPONSE_UP	-0.44	-1.72	0.00	0.01
HALLMARK_CHOLESTEROL_HOMEOSTASIS	-0.48	-1.72	0.00	0.01
HALLMARK_OXIDATIVE_PHOSPHORYLATION	-0.36	-1.52	0.00	0.04
HALLMARK_MTORC1_SIGNALING	-0.35	-1.48	0.00	0.05
HALLMARK_APICAL_JUNCTION	-0.36	-1.42	0.01	0.07
HALLMARK_MITOTIC_SPINDLE	-0.34	-1.42	0.00	0.07
HALLMARK_ESTROGEN_RESPONSE_LATE	-0.34	-1.38	0.01	0.09
HALLMARK_TNFA_SIGNALING_VIA_NFKB	-0.34	-1.37	0.02	0.09
HALLMARK_ESTROGEN_RESPONSE_EARLY	-0.33	-1.34	0.03	0.11
HALLMARK_GLYCOLYSIS	-0.33	-1.34	0.03	0.10
HALLMARK_PI3K_AKT_MTOR_SIGNALING	-0.36	-1.31	0.07	0.12

Suppl. Table S6

LAPC4.daroHigh.R1881.22h

DOWN-regulated pathways

NAME	ES	NES	NOM p-val	FDR q-val
HALLMARK_ANDROGEN_RESPONSE	-0.74	-2.57	0.00	0.00
HALLMARK_MYC_TARGETS_V2	-0.74	-2.37	0.00	0.00
HALLMARK_ESTROGEN_RESPONSE_EARLY	-0.53	-1.98	0.00	0.00
HALLMARK_KRAS_SIGNALING_DN	-0.58	-1.97	0.00	0.00
HALLMARK_E2F_TARGETS	-0.51	-1.93	0.00	0.00
HALLMARK_ESTROGEN_RESPONSE_LATE	-0.50	-1.84	0.00	0.00
HALLMARK_G2M_CHECKPOINT	-0.44	-1.68	0.00	0.02
HALLMARK_HEDGEHOG_SIGNALING	-0.61	-1.61	0.03	0.03
HALLMARK_MYC_TARGETS_V1	-0.41	-1.56	0.00	0.04
HALLMARK_WNT_BETA_CATENIN_SIGNALING	-0.52	-1.52	0.03	0.06
HALLMARK_BILE_ACID_METABOLISM	-0.45	-1.50	0.03	0.07
HALLMARK_REACTIVE_OXIGEN_SPECIES_PATHWAY	-0.50	-1.47	0.04	0.07
HALLMARK_MTORC1_SIGNALING	-0.38	-1.47	0.01	0.07
HALLMARK_UNFOLDED_PROTEIN_RESPONSE	-0.40	-1.40	0.04	0.10
HALLMARK_FATTY_ACID_METABOLISM	-0.36	-1.30	0.05	0.21
HALLMARK_CHOLESTEROL_HOMEOSTASIS	-0.40	-1.30	0.09	0.20
HALLMARK_XENOBIOTIC_METABOLISM	-0.36	-1.29	0.07	0.19

UP-regulated pathways

NAME	ES	NES	NOM p-val	FDR q-val
HALLMARK_COAGULATION	0.41	1.41	0.02	0.32
HALLMARK_INFLAMMATORY_RESPONSE	0.38	1.40	0.02	0.18
HALLMARK_COMPLEMENT	0.36	1.38	0.04	0.14

Suppl. Table S7

LAPC4.daroHigh.R1881.8h

DOWN-regulated pathways

NAME	ES	NES	NOM p-val	FDR q-val
HALLMARK_ANDROGEN_RESPONSE	-0.69	-2.31	0.00	0.00
HALLMARK_ESTROGEN_RESPONSE_EARLY	-0.48	-1.74	0.00	0.02
HALLMARK_KRAS_SIGNALING_DN	-0.46	-1.51	0.02	0.17

UP-regulated pathways

NAME	ES	NES	NOM p-val	FDR q-val
HALLMARK_INTERFERON_ALPHA_RESPONSE	0.50	1.70	0.01	0.03
HALLMARK_INFLAMMATORY_RESPONSE	0.40	1.43	0.03	0.18
HALLMARK_APOPTOSIS	0.37	1.40	0.02	0.15

Suppl. Table S8

LAPC4.daroLow.R1881.22h

DOWN-regulated pathways

NAME	ES	NES	NOM p-val	FDR q-val
HALLMARK_ANDROGEN_RESPONSE	-0.76	-2.71	0.00	0.00
HALLMARK_MYC_TARGETS_V2	-0.71	-2.29	0.00	0.00
HALLMARK_MYC_TARGETS_V1	-0.55	-2.15	0.00	0.00
HALLMARK_E2F_TARGETS	-0.54	-2.12	0.00	0.00
HALLMARK_ESTROGEN_RESPONSE_LATE	-0.50	-1.90	0.00	0.00
HALLMARK_KRAS_SIGNALING_DN	-0.53	-1.86	0.00	0.00
HALLMARK_G2M_CHECKPOINT	-0.45	-1.77	0.00	0.00
HALLMARK_ESTROGEN_RESPONSE_EARLY	-0.46	-1.76	0.00	0.00
HALLMARK_MTORC1_SIGNALING	-0.43	-1.65	0.00	0.02
HALLMARK_REACTIVE_OXIGEN_SPECIES_PATHWAY	-0.52	-1.57	0.02	0.03
HALLMARK_UNFOLDED_PROTEIN_RESPONSE	-0.42	-1.52	0.01	0.04
HALLMARK_HEDGEHOG_SIGNALING	-0.51	-1.36	0.11	0.13
HALLMARK_FATTY_ACID_METABOLISM	-0.36	-1.34	0.04	0.14
HALLMARK_BILE_ACID_METABOLISM	-0.40	-1.33	0.05	0.14
HALLMARK_XENOBIOTIC_METABOLISM	-0.35	-1.32	0.06	0.14
HALLMARK_WNT_BETA_CATENIN_SIGNALING	-0.44	-1.28	0.13	0.17
HALLMARK_ALLOGRAFT_REJECTION	-0.36	-1.26	0.11	0.18

UP-regulated pathways

NAME	ES	NES	NOM p-val	FDR q-val
HALLMARK_COAGULATION	0.49	1.61	0.00	0.06
HALLMARK_INTERFERON_ALPHA_RESPONSE	0.45	1.48	0.02	0.13
HALLMARK_INFLAMMATORY_RESPONSE	0.42	1.45	0.02	0.12
HALLMARK_APOPTOSIS	0.40	1.45	0.02	0.09
HALLMARK_EPITHELIAL_MESENCHYMAL_TRANSITION	0.40	1.44	0.02	0.07
HALLMARK_MYOGENESIS	0.39	1.39	0.02	0.11

Suppl. Table S9

LAPC4.daroLow.R1881.8h

DOWN-regulated pathways

NAME	ES	NES	NOM p-val	FDR q-val
HALLMARK_ANDROGEN_RESPONSE	-0.66	-2.18	0.00	0.00

UP-regulated pathways

NAME	ES	NES	NOM p-val	FDR q-val
HALLMARK_INTERFERON_ALPHA_RESPONSE	0.58	1.88	0.00	0.00
HALLMARK_IL6_JAK_STAT3_SIGNALING	0.57	1.67	0.00	0.02
HALLMARK_INTERFERON_GAMMA_RESPONSE	0.44	1.58	0.00	0.04
HALLMARK_COMPLEMENT	0.38	1.35	0.04	0.20

Suppl. Table S10

Chr	Start	End	Gene
chr1	8472513	8472976	RERE
chr1	8472513	8472976	RERE
chr1	8472513	8472976	RERE
chr1	188139084	188139373	
chr1	198806651	198807023	
chr1	217076053	217076461	ESRRG
chr1	217076053	217076461	ESRRG
chr1	217076053	217076461	ESRRG
chr1	217076053	217076461	ESRRG
chr1	217076053	217076461	ESRRG
chr1	217076053	217076461	ESRRG
chr1	217076053	217076461	ESRRG
chr1	217076053	217076461	ESRRG
chr1	217076053	217076461	ESRRG
chr1	217076053	217076461	ESRRG
chr1	217076053	217076461	ESRRG
chr1	217076053	217076461	ESRRG
chr1	217076053	217076461	ESRRG
chr1	217076053	217076461	ESRRG
chr1	217076053	217076461	ESRRG
chr10	34704358	34704676	PARD3
chr10	34704358	34704676	PARD3
chr10	34704358	34704676	PARD3
chr10	34704358	34704676	PARD3
chr10	34704358	34704676	PARD3
chr10	34704358	34704676	PARD3
chr10	34704358	34704676	PARD3
chr10	34704358	34704676	PARD3
chr10	34704358	34704676	PARD3
chr10	34704358	34704676	PARD3
chr10	34704358	34704676	PARD3
chr10	52339808	52340155	SGMS1
chr10	59519533	59519860	
chr10	82610220	82610550	
chr10	83029824	83030240	
chr10	110481580	110482075	
chr11	15992116	15992573	SOX6
chr11	15992116	15992573	SOX6
chr11	15992116	15992573	SOX6
chr11	15992116	15992573	SOX6
chr11	111008334	111009045	
chr11	114049823	114050471	ZBTB16
chr11	114049823	114050471	ZBTB16
chr11	114049823	114050471	ZBTB16
chr11	114049823	114050471	ZBTB16
chr11	114049823	114050471	ZBTB16
chr12	64983864	64984360	RASSF3
chr13	33802046	33802359	STARD13
chr13	33802046	33802359	STARD13
chr13	36665978	36666238	DCLK1
chr13	36665978	36666238	DCLK1
chr13	36665978	36666238	DCLK1
chr13	44792687	44793448	
chr13	77407566	77408006	
chr14	25423491	25423814	STXBP6
chr14	25423491	25423814	STXBP6
chr14	25423491	25423814	STXBP6
chr14	25423491	25423814	STXBP6
chr14	25423491	25423814	STXBP6
chr14	25423491	25423814	STXBP6
chr14	25423491	25423814	STXBP6
chr14	56355942	56356332	
chr14	58285252	58285617	SLC35F4
chr14	58285252	58285617	SLC35F4
chr14	58285252	58285617	SLC35F4
chr14	58285252	58285617	SLC35F4
chr14	58285252	58285617	SLC35F4
chr14	58285252	58285617	SLC35F4
chr14	58285252	58285617	SLC35F4
chr14	58285252	58285617	SLC35F4
chr14	81848898	81849487	STON2
chr14	81848898	81849487	STON2
chr14	90038917	90039221	FOXN3
chr15	81680640	81681074	TMC3
chr15	89672867	89673370	ABHD2
chr15	89672867	89673370	ABHD2

Chr	Start	End	Gene
chr16	17479004	17479395	XYLT1
chr17	60803118	60803580	MARCH10
chr17	60803118	60803580	MARCH10
chr17	60803118	60803580	MARCH10
chr17	60803118	60803580	MARCH10
chr17	74170072	74170595	RNF157
chr17	74170072	74170595	RNF157
chr19	4991556	4992545	KDM4B
chr19	13102090	13102519	NFIX
chr2	20995594	20995962	LDAH
chr2	20995594	20995962	LDAH
chr2	20995594	20995962	LDAH
chr2	20995594	20995962	LDAH
chr2	20995594	20995962	LDAH
chr2	20995594	20995962	LDAH
chr2	20995594	20995962	LDAH
chr2	23162253	23162708	
chr2	48522581	48522939	FOXN2
chr2	79493030	79493431	
chr2	124941012	124941398	CNTNAP5
chr2	139651336	139651689	
chr2	164224296	164224695	
chr2	180438827	180439363	ZNF385B
chr2	180438827	180439363	ZNF385B
chr2	180438827	180439363	ZNF385B
chr2	180438827	180439363	ZNF385B
chr2	180438827	180439363	ZNF385B
chr2	180438827	180439363	ZNF385B
chr2	180438827	180439363	ZNF385B
chr2	180438827	180439363	ZNF385B
chr2	180438827	180439363	ZNF385B
chr2	180438827	180439363	ZNF385B
chr2	180438827	180439363	ZNF385B
chr2	180438827	180439363	ZNF385B
chr2	180438827	180439363	ZNF385B
chr2	180438827	180439363	ZNF385B
chr2	181579919	181580210	
chr2	215259761	215260100	VWC2L
chr2	215259761	215260100	VWC2L
chr2	215259761	215260100	SPAG16
chr2	234990977	234991504	
chr20	8075013	8075555	
chr20	25260637	25261111	PYGB
chr20	37233313	37233915	ARHGAP40
chr3	72355993	72356378	
chr3	104317870	104318431	
chr3	152103542	152103906	MBNL1
chr3	152103542	152103906	MBNL1
chr3	152103542	152103906	MBNL1
chr3	152103542	152103906	MBNL1
chr3	152103542	152103906	MBNL1
chr3	152103542	152103906	MBNL1
chr3	152103542	152103906	MBNL1
chr3	152103542	152103906	MBNL1
chr3	174058715	174059184	
chr3	187924572	187925058	LPP
chr3	187924572	187925058	LPP
chr3	187924572	187925058	LPP
chr4	20640997	20641330	
chr4	169600945	169601253	PALLD
chr4	169600945	169601253	PALLD
chr4	169600945	169601253	PALLD
chr4	181509697	181510001	
chr5	14716886	14717543	ANKH
chr5	15356838	15357270	
chr5	16547548	16547971	RETREG1
chr5	42426683	42427063	GHR
chr5	42426683	42427063	GHR
chr5	42426683	42427063	GHR
chr5	42426683	42427063	GHR
chr5	147291049	147291467	C5orf46
chr6	1838918	1839267	GMDS
chr6	1838918	1839267	GMDS
chr6	23692515	23692900	

Chr	Start	End	Gene
chr6	34192274	34192724	HMGA1
chr6	34192274	34192724	HMGA1
chr6	34192274	34192724	HMGA1
chr6	34192274	34192724	HMGA1
chr6	34192274	34192724	HMGA1
chr6	34192274	34192724	HMGA1
chr6	34192274	34192724	HMGA1
chr6	34192274	34192724	HMGA1
chr6	34192274	34192724	HMGA1
chr6	34192274	34192724	HMGA1
chr6	34192274	34192724	HMGA1
chr6	34192274	34192724	HMGA1
chr6	49874602	49874910	
chr6	91379646	91379898	
chr6	110975281	110975887	CDK19
chr6	110975281	110975887	CDK19
chr6	110975281	110975887	CDK19
chr6	110975281	110975887	CDK19
chr6	139806823	139807230	
chr6	147500899	147501356	
chr7	14808960	14809330	DGKB
chr7	14808960	14809330	DGKB
chr7	14808960	14809330	DGKB
chr7	14808960	14809330	DGKB
chr7	14808960	14809330	DGKB
chr7	14808960	14809330	DGKB
chr7	14808960	14809330	DGKB
chr7	14808960	14809330	DGKB
chr7	14808960	14809330	DGKB
chr7	14808960	14809330	DGKB
chr7	14808960	14809330	DGKB
chr7	14808960	14809330	DGKB
chr7	14808960	14809330	DGKB
chr7	14808960	14809330	DGKB
chr7	14808960	14809330	DGKB
chr7	14808960	14809330	DGKB
chr7	14808960	14809330	DGKB
chr7	14808960	14809330	DGKB
chr7	14808960	14809330	DGKB
chr7	27975958	27976788	JAZF1
chr7	42109328	42110024	GLI3
chr7	81120862	81121266	
chr7	84183001	84183286	
chr7	104431528	104431885	LHFPL3
chr7	108251653	108252033	
chr7	111169647	111170531	IMMP2L
chr7	111169647	111170531	IMMP2L
chr7	111169647	111170531	IMMP2L
chr7	111169647	111170531	IMMP2L
chr7	111169647	111170531	IMMP2L
chr7	111169647	111170531	IMMP2L
chr7	111169647	111170531	IMMP2L
chr7	111169647	111170531	IMMP2L
chr7	124559890	124560322	POT1
chr7	124559890	124560322	POT1
chr7	125124817	125125264	
chr8	62946822	62947318	
chr8	97976286	97976650	CPQ
chr8	102435924	102436231	
chr8	134467829	134468304	ST3GAL1
chr8	134467829	134468304	ST3GAL1
chr9	22455292	22455641	
chr9	89020101	89020497	
chr9	112063341	112063938	EPB41L4B
chr9	112063341	112063938	EPB41L4B
chr9	140195282	140195643	NRARP
chrX	2596579	2597068	CD99
chrX	2596579	2597068	CD99
chrX	2596579	2597068	CD99
chrX	2596579	2597068	CD99
chrX	2596579	2597068	CD99
chrX	2596579	2597068	CD99
chrX	11482464	11482798	ARHGAP6
chrX	11482464	11482798	ARHGAP6
chrX	149253107	149253429	

Suppl. Table S11

VCaP

cluster 1 DMSO R1881 Daro

MSigDB Perturbation	log10(Binom. p-val.)
Genes up-regulated in LNCAP cells in response to synthetic androgen R1881	28.66
Genes up-regulated in MDA-MB-453 cells (class A ER(-)) after exposure to the androgen R1881	15.58
Genes up-regulated in prostate cancer samples	13.99
Up-regulated genes in the left ventricle myocardium of patients with heart failure following implantation of LVAD	11.69
Genes up-regulated in hepatocellular carcinoma induced by ciprofibrate	10.94

MSigDB Pathway	log10(Binom. p-val.)
FOXA1 transcription factor network	9.92
Genes involved in NOTCH1 intracellular domain regulated transcription	9.85
Notch-mediated HES/HEY network	7.99
Mechanism of gene regulation by PPARa(alpha)	7.91

cluster 2 R1881 Daro

MSigDB Perturbation	log10(Binom. p-val.)
Genes up-regulated in LNCAP cells in response to synthetic androgen R1881	5.36
Genes up-regulated in H1975 cells resistant to gefitinib after treatment with EGFR inhibitor for 24h	5.11
Up-regulated genes in the left ventricle myocardium of patients with heart failure following implantation of LVAD	5.04
Cluster 9: genes up-regulated in SW260 cells by sodium butyrate and TSA with the same kinetics with which each alters the level of histone H4 acetylation.	4.97
Genes up-regulated in constitutively invasive HT-29 5M21 cells vs those expressing functionally inactive TATI	4.87

MSigDB Pathway	log10(Binom. p-val.)
TGF-beta receptor signaling	5.4

cluster 3 R1881 DMSO

MSigDB Pathway	log10(Binom. p-val.)
FOXA1 transcription factor network	6

cluster 4 R1881

MSigDB Perturbation	log10(Binom. p-val.)
Top genes up-regulated in liver tissue from mice with knockout of ZMPSTE24	27.47
Genes down-regulated in 3T3-L1 cells (adipocyte) by insulin but displayed blunted response to insulin the insulin resistant cells	11.34
Classic adipogenic genes (group1) that are induced by PPARG during adipogensis in 3T3-L1 preadipocytes	9.71
STAT3 targets in hematopoietic signaling	9.69
Genes up-regulated in U937 cells (acute promyelocytic leukemia, APL) by retinoin (ATRA)	9.33

cluster 5 DMSO

MSigDB Pathway	log10(Binom. p-val.)
Genes involved in downstream signal transduction	5.41
Validated targets of C-MAX transcriptional repression	4.72
Acute myeloid leukemia	4.18
Genes in prolonged ERK activation events	3.66
Class I PI3K signaling events mediated by Akt	3.63
Genes involved in PI3K/AKT activation	3.21

LAPC4

cluster 4 R1881

MSigDB Perturbation	log10(Binom. p-val.)
Genes up-regulated in LNCAP cells in response to synthetic androgen R1881	18.65
Genes up-regulated in MDA-MB-453 cells (class A ER(-) after exposure to the androgen R1881	18.05
Genes up-regulated in prostate tumor vs normal tissue samples	9
Genes up-regulated in ER(-) / PR(-) breast tumors with molecular similarity to ER(+) (class A) relative to the rest of the ER(-) / PR(-) samples	9.74
Genes down-regulated in HMLE cells after loss of function of CDH1, which was achieved either by RNAi knockdown or by expression of a dominant-negative form of CDH1	7.56

Suppl. Table S12

VCaP - SE associated:

	Disease Ontology	log10 binom p val
1	Thyroid Gland Disease	7.16
2	Prostate Disease	6.89
3	Neck Cancer	6.7
4	Neck Neoplasm	6.66
5	Endometrial Carcinoma	6.58
6	Cancer of Urinary Tract	5.98
7	Gastrointestinal Neoplasm	5.83
8	Kidney Neoplasm	5.74
9	Digestive System Cancer	5.64
10	Prostatic Neoplasm	5.54
11	Bladder Disease	5.52

	MSigDB Perturbation	log10 binom p val
1	Genes up-regulated in LNCAP cells in response to synthetic androgen R1881	17.56
2	Genes up-regulated in luminal-like breast cancer cell lines compared to the mesenchymal-like ones	9.59
3	Genes up-regulated in poorly differentiated thyroid carcinoma compared to normal thyroid tissue	9.24
4	The group 5 set of genes associated with acquired endocrine therapy resistance in breast tumors expressing ESR1 but not ERBB2	8.26
5	Class I of genes transiently induced by EGF in 184A1 cells	7.95
6	Genes up-regulated in prostate cancer samples	7.79
7	Up-regulated genes in the left ventricle myocardium of patients with heart failure	7.71
8	Genes up-regulated in hepatocellular induced by ciprofibrate	7.63
